# User Engagement With mHealth Interventions to Promote Treatment Adherence and Self-Management in People With Chronic Health Conditions: Systematic Review

**DOI:** 10.2196/50508

**Published:** 2024-09-24

**Authors:** Cyd Eaton, Natalie Vallejo, Xiomara McDonald, Jasmine Wu, Rosa Rodríguez, Nishanth Muthusamy, Nestoras Mathioudakis, Kristin A Riekert

**Affiliations:** 1 Johns Hopkins School of Medicine Baltimore, MD United States

**Keywords:** mobile health, mHealth, digital health, treatment adherence, self-management, user engagement, chronic health conditions, mobile phone

## Abstract

**Background:**

There are numerous mobile health (mHealth) interventions for treatment adherence and self-management; yet, little is known about user engagement or interaction with these technologies.

**Objective:**

This systematic review aimed to answer the following questions: (1) How is user engagement defined and measured in studies of mHealth interventions to promote adherence to prescribed medical or health regimens or self-management among people living with a health condition? (2) To what degree are patients engaging with these mHealth interventions? (3) What is the association between user engagement with mHealth interventions and adherence or self-management outcomes? (4) How often is user engagement a research end point?

**Methods:**

Scientific database (Ovid MEDLINE, Embase, Web of Science, PsycINFO, and CINAHL) search results (2016-2021) were screened for inclusion and exclusion criteria. Data were extracted in a standardized electronic form. No risk-of-bias assessment was conducted because this review aimed to characterize user engagement measurement rather than certainty in primary study results. The results were synthesized descriptively and thematically.

**Results:**

A total of 292 studies were included for data extraction. The median number of participants per study was 77 (IQR 34-164). Most of the mHealth interventions were evaluated in nonrandomized studies (157/292, 53.8%), involved people with diabetes (51/292, 17.5%), targeted medication adherence (98/292, 33.6%), and comprised apps (220/292, 75.3%). The principal findings were as follows: (1) >60 unique terms were used to define user engagement; “use” (102/292, 34.9%) and “engagement” (94/292, 32.2%) were the most common; (2) a total of 11 distinct user engagement measurement approaches were identified; the use of objective user log-in data from an app or web portal (160/292, 54.8%) was the most common; (3) although engagement was inconsistently evaluated, most of the studies (99/195, 50.8%) reported >1 level of engagement due to the use of multiple measurement methods or analyses, decreased engagement across time (76/99, 77%), and results and conclusions suggesting that higher engagement was associated with positive adherence or self-management (60/103, 58.3%); and (4) user engagement was a research end point in only 19.2% (56/292) of the studies.

**Conclusions:**

The results revealed major limitations in the literature reviewed, including significant variability in how user engagement is defined, a tendency to rely on user log-in data over other measurements, and critical gaps in how user engagement is evaluated (infrequently evaluated over time or in relation to adherence or self-management outcomes and rarely considered a research end point). Recommendations are outlined in response to our findings with the goal of improving research rigor in this area.

**Trial Registration:**

PROSPERO International Prospective Register of Systematic Reviews CRD42022289693; https://www.crd.york.ac.uk/prospero/display_record.php?ID=CRD42022289693

## Introduction

### Background

As smartphones have become an integral part of modern daily life [1,2], mobile health (mHealth) interventions for treatment adherence and self-management promotion have rapidly developed. These interventions often use existing smartphone features, such as sending SMS text notifications as cues to take prescribed medications [3]; others may integrate external technologies, such as a Bluetooth-enabled glucometer linked to a smartphone app to track blood glucose levels over time with the goal of supporting diabetes management [4]. In general, more frequent engagement, or interaction, with mHealth tools is expected to result in improved treatment adherence or self-management [5]. However, mHealth tools are frequently abandoned by users. Among mobile phone users in the United States, more than half reportedly downloaded an mHealth app but nearly half also stopped using the app due to high data entry burden, low interest, and costs [6].

There is a disconnect between research findings supporting positive correlations between engagement and adherence or self-management and user tendencies to stop using mHealth tools. This discrepancy may reflect an argument put forth by Arigo et al [7] that the mHealth field “lacks a science of engagement.” Specifically, there is (1) a lack of consensus in how mHealth user engagement is measured, defined, and reported; (2) no consensus on the optimal level of mHealth user engagement to facilitate meaningful behavior change; and (3) infrequent consideration of user engagement as a research end point. Not treating user engagement as a research end point suggests that this domain is poorly defined and haphazardly evaluated, particularly in terms of how user engagement might evolve over the course of the intervention or relate to behavioral and health outcomes. While the tendency for mHealth use to decline over time suggests that users will not experience maximum benefit from accessing these tools, poor measurement of user engagement has presented a challenge to researchers’ abilities to characterize exactly how and why engagement may decrease and how these decreases may affect intervention outcomes. These critical gaps in the science of user engagement significantly limit the utility, effectiveness, uptake, and scalability of mHealth interventions for adherence and self-management promotion.

Recent systematic reviews have examined aspects of user engagement with mHealth interventions for specific diagnoses, including hypertension [8], physical activity [9,10], depressive symptoms [11,12], and mental health conditions [13]. The applicability of these reviews to adherence and self-management mHealth interventions is significantly limited by the small number of studies included and a lack of unified focus on adherence and self-management behaviors. These prior reviews have been further limited by using a very broad definition of *engagement* to include usability, feasibility, user satisfaction, and acceptability [13], limiting the review to studies in which only postassessment retention data were obtained [11] (excludes interventions earlier in the design phase), and focusing on design features associated with user engagement rather than the evaluation of user engagement itself [10]. Another review identified a range of valid and reliable measurement approaches for evaluating user engagement with mHealth interventions for behavior change but used a snowballing method to identify sources rather than a rigorous systematic review of the literature [14]. To encourage the continued use of, and optimal engagement with, mHealth interventions to facilitate adherence and self-management behavior change, it is imperative to comprehensively and systematically evaluate the recent scientific landscape of mHealth user engagement with a clear focus on adherence and self-management behavior.

### Objectives

In response to gaps identified in the current scientific literature [7], our registered systematic review aimed to (1) characterize user engagement with interventions promoting mHealth treatment adherence or self-management for adults and youth with health conditions and (2) generate user engagement–focused research recommendations. “User engagement” with mHealth tools can be conceptualized as both behavioral (the extent of use) and experiential (the subjective experience of interacting with the technology) [15]. To enhance the practical application of our review findings, we focused on the behavioral aspects of user engagement to evaluate the degree of use and interaction with the mHealth tool [7] among users with chronic health conditions. Users’ behavioral interaction with mHealth tools, features, and associated behavior change components is known as “Little e” engagement. In theory, increased “Little e” engagement is expected to contribute to increased engagement in the desired health behavior, known as “Big E” [16]. Thus, better precision in how behavioral interaction, or “Little e” engagement, is empirically evaluated could help to facilitate greater changes in “Big E” outcomes, thus improving the overall efficacy of mHealth interventions for adherence and self-management. A systematic review approach was selected due to expected heterogeneity in both the measurement of user engagement and adherence and self-management outcomes, which precludes the use of a meta-analysis [17-19]. We specifically aimed to answer the following research questions: (1) How is user engagement defined and measured in studies of mHealth interventions to promote adherence to prescribed medical or health regimens or self-management among people living with a health condition? (2) To what degree are patients engaging with these mHealth interventions? (3) What is the association between user engagement with mHealth interventions and adherence or self-management outcomes? (4) How often is user engagement a research end point?

We also developed the following exploratory question: are there differences in user engagement measurement approaches and levels between studies that provide monetary compensation and those that do not?

## Methods

This systematic review was registered with PROSPERO (CRD42022289693) and prepared in accordance with the PRISMA (Preferred Reporting Items for Systematic Reviews and Meta-Analyses) guidelines. The review team prepared and followed a standard manual of procedures designed for this systematic review.

### Ethical Considerations

Institutional review board approval was not required because the study was not considered human participant research.

### Information Sources and Search Strategy

The search strategy (Multimedia Appendix 1) was developed by the first and last authors (CE and KR) in collaboration with an informationist at Johns Hopkins Libraries; implemented in Ovid MEDLINE, Embase, Web of Science, PsycINFO, and CINAHL; and restricted to manuscripts published between 2016 and 2021. All citations returned from the search were imported into our Covidence (Veritas Health Innovation Ltd) [20] database for screening and data extraction.

### Eligibility Criteria

The inclusion and exclusion criteria are presented in Textbox 1.

**Inclusion and exclusion criteria.**

**Inclusion criteria**

- Peer-reviewed manuscripts reporting on original qualitative or quantitative investigations published in English between 2016 and 2021
- Participants followed a medical or health regimen or engaged in adherence or self-management activities for a chronic physical or mental health condition
- A mobile health (mHealth) intervention was used by these participants or their caregivers in a home setting, was at least partially automated (could not only include manual 2-way SMS text messaging or video web conferencing), and was accessible on a mobile device (smartphone or tablet device, including internet browser–based programs)
- The primary intervention target was treatment adherence (eg, taking medication, exercising, or following a diet); or self-management of the health condition, often measured by a health outcome associated with treatment adherence behavior (eg, glycated hemoglobin test, viral load, or BMI)
- User engagement (use and interaction) with the mHealth intervention was a measured study outcome, either by objective (eg, app-recorded log-in data) or subjective (eg, user self-report or qualitative interviews) metrics

**Exclusion criteria**

- Meta-analyses, systematic reviews, published abstracts, dissertations, and published protocols, as well as studies reporting on usability testing or intervention development only

### Selection Process

Citations were imported into Covidence; duplicate citations were removed; and title and abstract screening was conducted, followed by a full-text review. At each level of review, 2 separate review team members evaluated each article against the inclusion and exclusion criteria, with discrepancies resolved by CE and N Muthusamy. Any study meeting the inclusion criteria after the full-text review progressed to the data extraction phase (detailed in the next subsection).

### Data Extraction

#### Overview

Data extraction was performed by 2 separate review team members independently of each other using a standard data extraction form developed by the study team, with discrepancies resolved by CE and N Muthusamy. For each study, the reviewers recorded both quantitative and qualitative data relevant to study design (eg, randomized controlled trial and case-control) and methodology (eg, monetary compensation for participation); demographic characteristics of participants; mHealth intervention targets and characteristics; measurement of user engagement, including whether it was a research end point (a key outcome being measured and potentially impacted by participation in the intervention); and the terminology used to describe the behavioral aspects of user engagement (researcher-evaluated user interactions with the mHealth technology).

Study results were summarized as described in the following subsections.

#### Level of Engagement With the Intervention

This was categorized as “high,” “medium,” “low,” “>1 level reported due to the use of multiple measurement or analytic approaches,” or “not characterized.” Categorizations were assigned based on the language used by the authors to characterize users’ level of engagement with the mHealth intervention (eg, the authors described user engagement with the mHealth intervention as “high”).

#### Change in Level of Engagement

This was categorized as “increased,” “no change,” “decreased,” “>1 direction reported due to the use of multiple measurement or analytic approaches,” or “not assessed.” For studies that assessed change in engagement over time, categorizations were assigned based on the data presented by the authors (eg, the authors presented data showing that the user engagement measurement decreased over time).

#### Association With Treatment Adherence or Self-Management Study Outcomes

This was categorized as “higher engagement, positive treatment adherence or self-management outcomes”; “moderate engagement, positive treatment adherence or self-management outcome)”; “lower engagement, positive treatment adherence or self-management outcomes”; “no association”; “>1 association reported due to the use of multiple measurement or analytic approaches”; or “not assessed.” For studies that assessed this association, categorizations were assigned based on how the authors reported and framed the study results and conclusions (eg, the authors’ reporting and framing of study results and conclusions suggested that higher engagement with the mHealth intervention was associated with positive study outcomes, such as higher treatment adherence or improved self-management outcome or outcomes).

#### Technology Dosage

This was categorized as “yes, given” (the researchers told participants how often or in what way or ways they should use the mHealth intervention components, such as complete 1 module per week and log medication administration in the app every day) or “no, not given.”

#### Minimum Engagement Research Benchmark

This was categorized as “yes, selected” (the researchers reported in their manuscript that a minimum cutoff for technology engagement was set as an empirical outcome to denote adequate participant engagement; eg, to be adequately engaged, a participant needed to use the Bluetooth-enabled glucometer at least once a day during the study period) or “no, not selected.”

### No Formal Risk-of-Bias Assessment

We decided not to conduct a formal risk-of-bias assessment, given that the primary aim of this review was to characterize the evaluation and measurement of user engagement rather than certainty in the primary study results. Therefore, it was deemed inappropriate to evaluate the studies using standard risk-of-bias assessment tools.

### Synthesis Methods

Statistical analyses were conducted using SPSS software (version 28.0; IBM Corp) [21]. The extracted data were summarized using frequencies and percentages. Methods of measuring user engagement with the mHealth intervention were thematically grouped into discrete measurement categories. Subgroup sensitivity analyses were conducted to examine studies involving pediatric samples (participants aged 0-18 y or aged up to 25 y if the sample was characterized as “pediatric” by the authors) separately from those involving adults only (participants aged >18 y). In these age-based subgroup analyses, of the 292 included studies, 5 (1.7%) were excluded due to including both pediatric and adult participants, and 1 (0.3%) was excluded due to not reporting participant ages. Given that this investigation was designed as a systematic review, no effect measures or meta-regressions were used. No missing summary statistics or data conversions were used.

## Results

### Search Results and Screening Process

**Figure 1:**
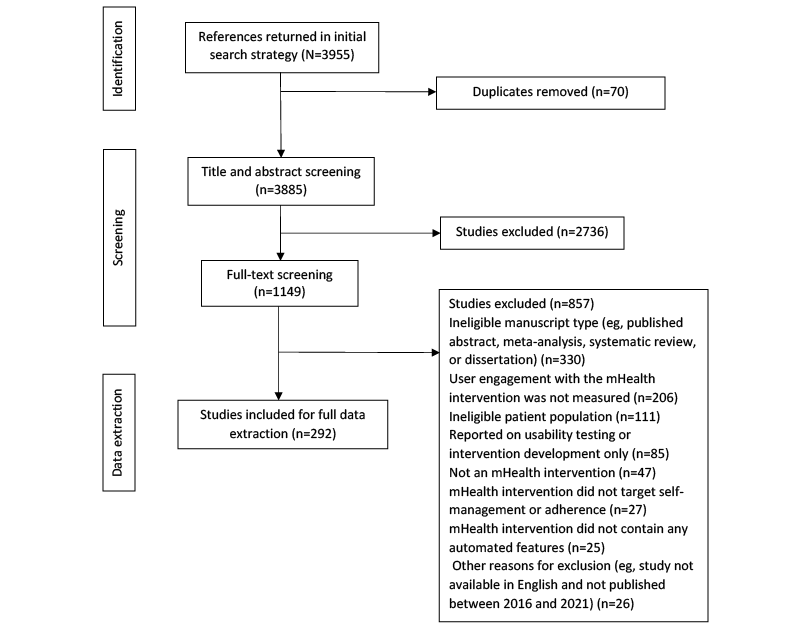
PRISMA (Preferred Reporting Items for Systematic Reviews and Meta-Analyses) flow diagram of the screening process. mHealth: mobile health.

Figure 1 presents the PRISMA flow diagram of the screening process. The initial search returned 3955 citations, from which 70 (1.77%) duplicates were removed. During title and abstract screening, the remaining 3885 studies were screened, and 2736 (70.42%) were excluded. During full-text screening, the remaining 1149 studies were evaluated, and 857 (74.59%) were excluded. The primary reasons for exclusion were as follows: ineligible manuscript type (eg, published abstract; 330/857, 38.5%), user engagement with the mHealth intervention was not measured (206/857, 24%), and ineligible participant population (111/857, 12.9%). The final review included 292 studies [22-313] (refer to Multimedia Appendix 2 for all included studies and characteristics).

### Basic Study and mHealth Intervention Characteristics

Nearly half of the studies (135/292, 46.2%) were conducted in the United States and used a randomized controlled trial design. The median number of participants was 77 (IQR 34-164). Nearly half (135/292, 46.2%) were considered feasibility studies. The median study length was 90 (IQR 60-180) days. Diabetes (51/292, 17.5%) and mental health conditions (35/292, 11.9%) were the most common diagnoses. Study characteristics were similar between adult and pediatric studies, with the exception of health conditions, reflecting expected age-based differences in diagnoses more common among adults than among children (eg, type 2 diabetes and substance use were more commonly studied in adult samples than in pediatric samples).

Table S1 in Multimedia Appendix 3 contains specific details on the mHealth interventions’ adherence or self-management targets, intervention components, and intended users. The most frequently targeted adherence or self-management concern was taking medication (98/292, 33.6%), followed by exercise (93/292, 31.8%) and diet (73/292, 25%). Nearly all mHealth interventions were used by the patient (291/292, 99.7%), but some of them included health care providers (58/292, 19.9%) or caregivers (20/292, 6.8%). The majority of mHealth interventions comprised a mobile app (220/292, 75.3%), SMS text messaging or push notifications (74/292, 25.3%), websites or web portals not within a mobile app (48/292, 16.4%), and nonwearable monitoring devices (47/292, 16.1%). Interventions that did not include a mobile app primarily comprised text messaging, a wearable device, a nonwearable device, video web conferencing or telephone calls, or a website or web portal not within a mobile app. Nearly three-quarters of the interventions (216/292, 74%) prompted users to engage with the mHealth intervention. Less than half of the studies (124/292, 42.5%) provided monetary compensation for participation. Only 10.6% (31/292) allowed users to continue using the mHealth intervention after the formal study period. Intervention characteristics were generally similar between adult and pediatric studies, with the exception of intervention target behavior, reflecting expected age-based differences in health concerns more common among adults than among children (eg, exercise, mental health management, and drug or alcohol use or abuse were more commonly targeted in adult samples than in pediatric samples).

### How Is User Engagement Defined and Measured?

#### Definition

Terminology defining user engagement varied widely (Table S2 in Multimedia Appendix 3). There were 33 unique terms used to define engagement that appeared in at least 2 (0.7%) of the 292 studies. Of the 292 studies, 31 (10.6%) studies each used a unique term that appeared in only that 1 study. “Use” (102/292, 34.9%) and “engagement” (94/292, 32.2%) were the most common terms. Although most terms were synonymous with “use,” “engagement,” or “interaction” with the technology (reflecting our a priori definition of behavioral user engagement and our search strategy), other studies notably used disparate terms, including “acceptability” (26/292, 8.9%), “fidelity” (5/292, 1.7%), “satisfaction” (6/292, 2%), and “perception” (6/292, 2%).

#### Measurement

Across all studies, 11 distinct user engagement measurement approaches emerged, comprising both objective (n=9, 82%) and subjective (n=2, 18%) methods. User engagement was most frequently evaluated via objective user log-in data from the app or web portal (eg, number of log-ins; 160/292, 54.8%), followed by manually entering data in an app (77/292, 26.4%), qualitative interviews (54/292, 18.5%), and responding to text notifications (49/292, 16.8%). There were “other objective measures” that did not fall into any of the 11 main categories (15/292, 5.1%; eg, notification reading rate or downloading podcasts). These results were similar between adult and pediatric studies (Table 1; Table S3 in Multimedia Appendix 3).

**Table 1:** How is user engagement measured (n=292)?

Measurement methods			Examples		Studies, n (%)^a^
**Objective measures**					
	User log-in data retrieved from app or website	Number of log-ins to app or website Length of time spent in app or website Frequency of accessing specific features within the app or website		160 (54.8)	
	Manual user data entry in app-or website-based self-monitoring diaries	User manually enters data in the app, such as blood glucose level, date and time when medicine was taken, or blood pressure values		77 (26.4)	
	Response to SMS text messages or push notifications	User types and sends a response to a SMS text message asking if they took their medicine that day		49 (16.8)	
	Number or proportion of intervention program modules completed within app or website	User completes 3 out of 6 possible modules on pain management skills		48 (16.4)	
	Interacting via chats, phone calls, or social media posts	Number of times user sends a chat message to care team through app		33 (11.3)	
	Wearing an electronic monitoring device	Length of time the user wore a Fitbit device to track daily step count		26 (8.9)	
	Using a nonwearable electronic monitoring device	Medication adherence is monitored with an electronic pill bottle that tracks when the bottle is opened and closed to administer medicine		26 (8.9)	
	Submitting videos via app	Medication adherence is measured using a mobile app designed to directly observe therapy		5 (1.7)	
	Other objective measures	Notification message reading rate or downloading podcasts		13 (4.5)	
**Subjective measures**					
	Qualitative interview	User completes a qualitative interview about their experience using the mHealth^b^ app		54 (18.5)	
	Participant-reported survey	User self-reports frequency of using the app		29 (9.9)	

^a^Percentages do not add up to 100% because studies could fall into >1 category.

^b^mHealth: mobile health.

When examining engagement definitions by measurement approaches, “use” (7/9, 78%) and “engagement” (9/9, 100%) were most commonly used across the nine objective measurement approaches. The exceptions were “wearing an electronic monitoring device” and “submitting videos via app” for which “adherence” (11/26, 42%, 2/5, 40%, respectively) was most commonly used within the measurement approach. Qualitative interviews had the widest range of terminologies used, with “user experience” being the most common (21/54, 39%; Table 2).

**Table 2:** Associations between user engagement evaluation methods and definitions.

Evaluation methods	Terms^a^ used to define user engagement, n (%)^b^
User log-in data retrieved from app or website (n=160)	Use: 78 (49) Engagement: 62 (39) Feasibility: 27 (17) Adherence: 25 (16)
Manual user data entry in app- or website-based self-monitoring diaries (n=77)	Use: 31 (40) Engagement: 24 (31) Adherence: 19 (25) Feasibility: 17 (22) Compliance: 10 (13)
Response to SMS text messages or push notifications (n=49)	Engagement: 21 (43) Response: 19 (39)
Number or proportion of intervention program modules completed within app or website (n=48)	Use: 22 (46) Engagement: 12 (25) Adherence: 11 (23)
Interacting via chats, phone calls, or social media posts (n=33)	Engagement: 14 (42) Use: 13 (39) Feasibility: 5 (15) Compliance: 4 (12)
Wearing an electronic monitoring device (n=26)	Adherence: 11 (42) Engagement: 9 (35) Feasibility: 6 (23)
Using a nonwearable electronic monitoring device (n=26)	Use: 9 (25) Adherence: 8 (31) Engagement: 6 (23)
Submitting videos via app (n=5)	Adherence: 2 (40) Use: 2 (40) Compliance: 1 (20) Engagement: 1 (20) Acceptability: 1 (20)
Other objective measures (n=13)	Engagement: 7 (54) Feasibility: 2 (15) Use: 2 (15) Response: 2 (15)
Qualitative interview (n=54)	User experience: 21 (39) Engagement: 18 (33) Use: 17 (31) Acceptability: 13 (24) Feasibility: 8 (15) Adherence: 8 (15)
Participant-reported survey (n=29)	Use: 13 (45) Feasibility: 10 (24) Engagement: 6 (21) Acceptability: 6 (21) Adherence: 5 (17)

^a^We report terms used to describe user engagement in at least 10% of the studies using a given evaluation method; this cutoff was selected to enhance interpretability due to the wide range of terms used to describe user engagement (refer to Table S2 in Multimedia Appendix 3 for details of user engagement definitions).

^b^Percentages within categories do not add up to 100% because studies could fall into >1 category.

The use of user log-in data was the most common measurement method across mHealth intervention components, except for SMS text messaging or push notifications and wearable devices. When SMS text messaging or push notifications was an intervention component, response to SMS text messaging or push notifications was the most common metric (38/74, 51%). When a wearable device was an intervention component, wearing an electronic monitoring device was the most common metric (26/39, 67%; Table S4 in Multimedia Appendix 3).

### To What Degree Are Participants Engaging With These mHealth Interventions?

#### User Engagement Level

User engagement level was characterized in two-thirds of the reviewed studies (195/292, 66.8%), of which a little more than half (99/195, 50.8%) reported >1 level of engagement due to the use of multiple measurement methods or analyses. Only one-third of the studies (99/292, 33.9%) examined change in engagement over time; when it was examined, engagement tended to decrease (76/99, 77%). These results were similar between adult and pediatric studies (Table 3).

**Table 3:** Degree of engagement and association with treatment adherence or self-management.

Characteristic		All studies (n=292), n (%)	Adult studies (n=241), n (%)	Pediatric studies (n=45), n (%)
**User engagement level**				
	High	63 (21.6)	55 (22.8)	7 (15.6)
	Medium	6 (2.1)	4 (1.7)	2 (4.4)
	Low	27 (9.2)	19 (7.9)	6 (13.3)
	>1^a^ level reported	99 (33.9)	81 (33.6)	18 (40.0)
	Not characterized	97 (33.2)	82 (34.0)	12 (26.7)
**Change in user engagement level**				
	Increased	3 (1.0)	3 (0.01)	0 (0)
	No change	15 (5.1)	13 (5.4)	1 (2.2)
	Decreased	76 (26.0)	61 (25.3)	13 (28.9)
	>1 direction reported	5 (1.7)	4 (1.7)	1 (2.2)
	Not assessed	193 (66.1)	160 (66.4)	30 (66.7)
**Association with adherence or SM^b^ outcomes**				
	Higher engagement, positive adherence or SM outcomes	60 (20.5)	49 (20.3)	8 (17.8)
	Moderate engagement, positive adherence or SM outcomes	1 (0.3)	1 (0.4)	0 (0)
	Lower engagement, positive adherence or SM outcomes	1 (0.3)	1 (0.4)	0 (0)
	No association	18 (6.2)	15 (6.2)	3 (6.7)
	>1 association reported	23 (7.9)	18 (7.5)	5 (11.1)
	Not assessed	189 (64.7)	157 (65.1)	29 (64.4)

^a^Categories with “>1” finding reflect the use of multiple measurements or analyses, leading to multiple results in different directions (eg, for “Change in user engagement level,” engagement is shown to increase and decrease depending on the measurement used).

^b^SM: self-management.

#### Measurement Approach by User Engagement Level

Compared to studies characterized as having high user engagement, studies with low user engagement tended to measure (≥10% difference) engagement with user log-in data (19/27, 70% vs 36/63, 57%) and module completion (7/27, 26% vs 9/63, 14%). Compared to studies characterized as having low user engagement, studies with high user engagement tended to measure engagement with response to text notifications (14/63, 22% vs 1/27, 4%); interacting via chats, phone calls, or social media posts (13/63, 21% vs 1/27, 4%); wearing an electronic monitoring device (6/63, 10% vs 0); using a nonwearable electronic monitoring device (7/63, 11% vs 0); and qualitative interviews (12/63, 19% vs 2/27, 7%; Table 4).

**Table 4:** Measurement approach by user engagement level with intervention.

Measurement	Characterization of user engagement level with intervention				
	Low (n=27), n (%)^a^	Medium (n=6), n (%)^a^	High (n=63), n (%)^a^	>1 (n=99), n (%)^a^	Not characterized (n=97), n (%)^a^
User log-in data retrieved from app or website	19 (70)	5 (83)	36 (57)	57 (57)	43 (44)
Manual user data entry in app-or website-based self-monitoring diaries	8 (29)	2 (33)	15 (24)	29 (29)	23 (24)
Response to SMS text messages or push notifications	1 (4)	0 (0)	14 (22)	14 (14)	20 (21)
Number or proportion of intervention program modules completed within app or website	7 (26)	2 (33)	9 (14)	16 (16)	14 (14)
Interacting via chats, phone calls, or social media posts	1 (4)	0 (0)	13 (21)	10 (10)	9 (33)
Wearing an electronic monitoring device	0 (0)	0 (0)	6 (10)	9 (9)	11 (11)
Using a nonwearable electronic monitoring device	0 (0)	0 (0)	7 (11)	11 (11)	8 (8)
Submitting videos via app	2 (7)	0 (0)	0 (0)	2 (20)	1 (20)
Other objective measures	2 (7)	0 (0)	3 (5)	5 (5)	3 (3)
Qualitative interview	2 (7)	1 (17)	12 (19)	16 (16)	23 (24)
Participant-reported survey	1 (4)	2 (33)	4 (6)	10 (10)	12 (12)

^a^Percentages within categories do not add up to 100% because studies could fall into >1 category.

#### Technology Dosages and Minimum Engagement Research Benchmarks

Technology dosages denote when researchers provided participants with specific recommendations for mHealth intervention use (eg, log in to the app at least 3 times a week). Minimum engagement research benchmarks denoted when researchers set a minimum research cutoff for adequate participant engagement (eg, a participant who responded to ≥75% of SMS text messages during the study period was considered by the researchers to be adequately engaged with the mHealth intervention). A research benchmark could be set without giving a technology dosage and vice versa. Technology dosages were given less than half the time to participants by the researchers across all studies (119/292, 40.8%) and when examined by age group. Whether technology dosages were given or not, researchers characterized engagement level as “low” (14/27, 52% vs 13/27, 48%) and “high” (29/63, 46% vs 34/63, 54%) in relatively equal proportions (<10% difference; Tables 5 and 6).

**Table 5:** Study characteristics based on technology dosage and minimum engagement research benchmark.

		All studies (n=292), n (%)	Adult studies (n=241), n (%)	Pediatric studies (n=45), n (%)
**Technology dosage given to participants**				
	Yes	119 (40.8)	96 (39.8)	22 (48.9)
	No	173 (59.2)	145 (60.2)	23 (51.1)
**Minimum engagement research benchmark set**				
	Yes	81 (27.7)	71 (29.4)	10 (22.2)
	No	211 (72.3)	170 (70.5)	35 (77.8)

**Table 6:** Engagement levels based on technology dosage and minimum engagement research benchmark.

		Low (n=27), n (%)	Medium (n=6), n (%)	High (n=63), n (%)	>1 (n=99), n (%)	Not characterized (n=97), n (%)
**Dosage given to participants**						
	Yes	14 (52)	2 (33)	29 (46)	43 (43)	31 (32)
	No	13 (48)	4 (67)	34 (54)	56 (57)	66 (68)
**Minimum engagement research benchmark set**						
	Yes	11 (41)	2 (33)	15 (24)	38 (38)	15 (15)
	No	16 (59)	4 (67)	48 (76)	61 (62)	82 (85)

A minimum engagement research benchmark was set as the outcome criterion less than one-third of the time across all studies (81/292, 27.7%) and when examined by age group. When a minimum engagement research benchmark was set, researchers tended to characterize user engagement levels as “low” (11/27, 41%) rather than “high” (15/63, 24%). When no minimum engagement research benchmark was set, researchers tended to characterize user engagement as “high” (48/63, 76%) rather than “low” (16/27, 59%; Table 5).

Among studies that had a minimum engagement research benchmark (81/292, 27.7%), less than half (35/81, 43%) gave a technology dosage to participants. Among the studies that gave a technology dosage to participants (119/292, 40.8%), only 29.4% (35/119) also had a minimum engagement research benchmark.

Of the 292 studies, 35 (12%) gave both a technology dosage and set a minimum engagement research benchmark; these indices matched in 94% (33/35) of the studies. Of these 35 studies, 29 (83%) characterized engagement level, of which the majority (n=13, 45%) reported >1 level of engagement due to the use of multiple measurement methods or analyses.

When a minimum engagement research benchmark was set or a recommended technology dosage was given, engagement tended to be measured with user log-in data (48/81, 59% and 69/119, 58%, respectively), manual data entry (26/81, 32% and 32/119, 26.9%, respectively), or module completion (16/81, 20% and 21/119, 17.6%, respectively; Table 7).

**Table 7:** User engagement measurement approach when minimum engagement research benchmarks were set or technology dosages were given.

Measurements	User engagement level set by researchers	
	Minimum engagement research benchmark (n=81), n (%)^a^	Technology dosage given (n=119), n (%)^a^
User log-in data retrieved from app or website	48 (59.3)	69 (58.0)
Manual user data entry in app- or website-based self-monitoring diaries	26 (32.1)	32 (26.9)
Response to SMS text messages or push notifications	9 (11.1)	15 (12.6)
Number or proportion of intervention program modules completed within app or website	16 (19.8)	21 (17.6)
Interacting via chats, phone calls, or social media posts	12 (14.8)	14 (11.8)
Wearing an electronic monitoring device	10 (12.3)	18 (15.1)
Using a nonwearable electronic monitoring device	10 (12.3)	14 (11.8)
Submitting videos via app	2 (2.5)	5 (4.2)
Other objective measures	4 (4.9)	7 (5.9)
Qualitative interview	9 (11.1)	16 (13.4)
Participant-reported survey	1 (0.01)	12 (10.1)

^a^Percentages within categories do not add up to 100% because studies could fall into >1 category.

### What Is the Association Between User Engagement With mHealth Interventions and Adherence or Self-Management Outcomes?

The association between engagement and treatment adherence or self-management outcomes was only assessed in a little more than one-third of the studies (103/292, 35.3%). Among these 103 studies, 60 (58.3%) tended to report and frame results and conclusions to suggest that higher engagement was associated with positive adherence or self-management outcomes. These results were similar between adult and pediatric studies (Table 3).

### How Often Is User Engagement a Research End Point?

User engagement was a research end point in only 19.2% (56/292) of the reviewed studies, with similar results in adult (44/241, 18.3%) and pediatric (11/45, 24%) studies. Of these 56 studies, 30 (54%) used nonrandomized experimental designs, and 27 (48%) were feasibility studies. User engagement was typically defined as “engagement” (24/56, 43%), “adherence” (16/56, 29%), or “use” (16/56, 29%; Table S2 in Multimedia Appendix 3) and was most frequently measured with user log-in data (31/56, 55%) or manual data entry (22/56, 39%; Table S5 in Multimedia Appendix 3).

### Exploratory Question: What Is the Association Between Providing Study Participants With Monetary Compensation and User Engagement Level?

Whether study participants were provided monetary compensation for their participation or not (or if compensation was not reported), user engagement measurement methods were used in similar proportions (<10% difference). Similarly, user engagement levels were observed in similar proportions between studies that provided monetary compensation and those that did not (or did not report compensation; Table S6 in Multimedia Appendix 3).

Of the 292 studies, 10 (3.4%) included as an intervention component financial incentives for using the technology or meeting mHealth intervention goals. Of these 10 studies, 7 (70%) reported user engagement levels. Among these 7 studies, in 1 (14%), the user engagement level was characterized as “high”; in 1 (14%) as “medium”; and in 1 (14%), as “low”; and 4 (57%) reported >1 level due to multiple measurement or analytic approaches.

## Discussion

### Principal Findings

The principal findings relative to our specific research questions are provided in the following subsections.

### How Is User Engagement Defined and Measured in Studies of mHealth Interventions to Promote Adherence to Prescribed Medical or Health Regimens or Self-Management Among People Living With a Health Condition?

Terms used to describe user engagement outcomes were wide ranging, but the most commonly used were “use” (102/292, 34.9%) and “engagement” (94/292, 32.2%). Across all studies reviewed, 11 distinct user engagement measurement approaches were identified, comprising both objective and subjective methods. The most common methods were user log-in data from the app or web portal (160/292, 54.8%), manually entering data in an app (77/292, 26.4%), qualitative interviews (54/292, 18.4%), and responding to SMS text messages or push notifications (49/292, 16.8%).

### To What Degree Are Patients Engaging With These mHealth Interventions?

User engagement level was difficult to quantify because it was only characterized in two-thirds of the reviewed studies (195/292, 66.8%), of which a little more than half (99/195, 50.8%) reported >1 level of engagement due to the use of multiple measurement methods or analyses. Only one-third of the studies (99/292, 33.9%) evaluated change in engagement over time, which tended to decrease.

### What Is the Association Between User Engagement With mHealth Interventions and Adherence or Self-Management Outcomes?

Only one-third of the studies (103/292, 35.3%) evaluated the association between engagement and treatment adherence or self-management outcomes. When evaluated, the study authors tended to report and frame results and conclusions to suggest that higher engagement was associated with positive adherence or self-management outcomes.

### How Often Is User Engagement a Research End Point?

User engagement was rarely considered a research end point (56/292, 19.2%).

### Exploratory Question: Are There Differences in User Engagement Measurement Approaches and Levels in Studies That Provide Monetary Compensation Compared to Those That Do Not?

No; whether study participants were provided monetary compensation for their participation or not (or if compensation was not reported), user engagement methods were used in similar proportions, and user engagement levels were observed in similar proportions.

### Implications and Future Directions

Despite immense focus in both commercial and research sectors on using mHealth to support chronic illness treatment adherence and self-management [314-316], people do not remain engaged with mHealth interventions in the long term [6]. Our systematic review also found that user engagement tends to decline over time. Consistent with the “Little e, Big E framework” [16], mHealth intervention success hinges on the expectation that people will interact with the technology and thereby experience intended behavior changes that will lead to better overall health. Most importantly, our systematic review revealed critical limitations with how user engagement is defined and evaluated, which significantly impedes our ability to (1) communicate about this topic and (2) draw strong conclusions about how much user engagement is necessary to achieve desired behavior and health changes.

A principal finding of our review was that there is no agreed-upon definition of mHealth user engagement, which is a direct barrier to interdisciplinary communication about this topic. We found >60 terms used to define user engagement, even when limiting inclusion criteria to the behavioral evaluation of this concept [15]. While “use” (102/292, 34.9%) and “engagement” (94/292, 32.2%) were the most common terms, “adherence” (59/292, 20.2%) also appeared frequently. “Adherence” as a term for mHealth user engagement can be problematic because it is closely tied to treatment adherence [317], a key target of many of the mHealth interventions included in this systematic review. “Adherence” can apply to engaging with the mHealth technology as intended, such as logging in to an app [109] or responding to SMS text messages [243] rather than following a prescribed treatment regimen. Engaging with the technology can have a direct connection to treatment adherence (eg, using a Bluetooth-enabled blood pressure cuff reflects adherence to a key part of the hypertension management regimen as well as engaging with the mHealth technology [118,308]). However, such interchangeability and a lack of consistency in terminology will continue to be a barrier to communicating within and outside the field and add to existing challenges with defining and evaluating this domain. The extremely wide range of terms used to define user engagement likely reflects a major critique of mHealth research—that there is a lack of a science of engagement [7]. We encourage standardization in terminology used when the intention is to evaluate users’ behavioral engagement with mHealth technology, rather than treatment regimen, with the most common terms found in this systematic review: “use” or “engagement.” Integrating standard user engagement language in an internationally adopted clinical terminology system, such as the Systematized Nomenclature of Medicine–Clinical Terms, could help facilitate standardization efforts.

Another key finding was that the use of user log-in data was the default mHealth user engagement outcome, except for wearing a device when the intervention involved a wearable device component and responding to SMS text messages and push notifications when the intervention involved SMS text messaging and push notifications. Reliance on user log-in data over the other 10 major measurement approaches likely reflects that these data can be relatively straightforward to extract from a web portal. A strength of the use of user log-in data is that it may indicate more effortful and deliberate interactions with the technology (the user likely needs sufficient motivation to open and navigate an app) compared with, for example, quickly responding to a SMS text message or wearing a pedometer on the wrist. User log-in data may be a more accurate reflection of engagement behavior than self-report, which is potentially subject to social desirability and recall bias. The tendency for engagement assessed using user log-in data to be characterized as “low” may reflect the higher user burden of logging in to, and interacting with an, app, in addition to the overwhelming selection of this method for measuring user engagement. Reliance on user log-in data may bias interpretations of user engagement to favor higher quantities of engagement at the expense of higher-quality engagement (eg, infrequent access of a particularly effective app feature that users perceive as interesting, helpful, or motivating may be higher-quality engagement compared with frequent log-ins to the app homepage). Given the ubiquitous selection of user log-in data and the heterogeneity of adherence and self-management outcomes in this systematic review, it is not possible to evaluate which user engagement metric is best. In general, investigators should select the user engagement metric reflecting essential interactions with the mHealth intervention’s key technology needed to facilitate behavior and health changes.

We found that the exact degree of user engagement with the interventions reviewed in this study was difficult to estimate and that the association between user engagement and adherence or self-management outcomes were rarely evaluated. Both findings may relate to an important secondary finding that researchers rarely set a priori benchmarks for how much user engagement is considered scientifically adequate (no minimum engagement research benchmark set). Concerningly, when *no* minimum research benchmark was set, researchers tended to characterize user engagement as “high,” suggesting overoptimism about study results in the absence of any hypothesized lower boundary for what constituted minimally acceptable mHealth use. Setting cut points for how much user engagement is hypothesized to be adequate would also help to guide how much and in what ways users should interact with the mHealth intervention components to experience meaningful improvements in target outcomes. Matching technology dosages to minimum engagement research benchmarks, when possible, could inform whether engagement recommendations were met and allow researchers to evaluate whether exceeding expectations is associated with even greater improvement in behavior or health outcomes. Of note, studies with technology dosages or minimum engagement research benchmarks seemed to demand more from participants, as evidenced by the more common use of user log-in data and manual data entry to evaluate user engagement. User engagement burden should be considered when setting mHealth engagement expectations.

Setting minimum engagement research benchmarks could also account for variability in user engagement–level characterization depending on the metric selected and help to define what constitutes effective and meaningful engagement. Specifically, engagement assessed using user log-in data tended to be characterized as “low.” By contrast, responding to text notifications or interacting via chats, phone calls, or social media posts, which may be more socially rewarding and less effortful to complete, tended to be characterized as “high.” A similarly higher pattern of user engagement was observed in studies evaluating the use of wearable (6/63, 10% vs 0) and nonwearable devices (7/63, 11% vs 0), which is often part of the users’ regular self-management routines (eg, Bluetooth-enabled pill bottle or glucometer) or passively worn (eg, pedometer) and thus may be less burdensome to use. A higher SMS text message response rate and comparatively lower app log-in rate may both be minimally acceptable and potentially meaningful levels of engagement to effectively facilitate behavior or health change, particularly considering the level of effort and motivation involved in each type of interaction. Given the inconsistencies in how investigators characterize engagement levels, setting minimum engagement research benchmarks could inform whether user engagement is adequate across metrics and associated with key adherence and self-management outcomes. This approach could also improve precision in how engagement levels are characterized (ie, what level of engagement is considered “low” vs “high”?). Such efforts during the study planning phase could reduce the tendency to characterize user engagement at multiple levels due to the use of multiple metrics and analyses. Rethinking engagement levels as “adequate,” “acceptable,” “effective,” or “meaningful” versus “high” or “low” may better inform how much people should realistically use mHealth technology and evaluate whether engagement level is associated with behavior and health changes.

In the mHealth trials reviewed, an important secondary finding was that participants were rarely told how much they should aim to engage with the technology (no technology dosages). This is in contrast to drug trials in which participants are instructed to take a specific dosage of medication on a strict schedule and trials of in-person behavior change interventions (traditional therapy) in which participants are given therapy regimens and expectations for participation (eg, attend 12 therapy sessions and complete assigned homework each week of treatment) [11]. Although an early-phase research goal may be to see how much people interact with the technology in the absence of recommendations, an important future goal is to understand how much engagement is needed to maximize behavior and health changes. To this end, recommending in what ways and how much users should aim to engage with the technology is necessary. Giving technology dosages would also allow for experimental evaluation of different levels of recommended technology use to see what recommendation helps users achieve the most positive behavior and health changes. Furthermore, improvements in health and wellness can take time, and mHealth users may not observe immediate benefits to using mHealth interventions, which could contribute to premature abandonment of the tool. Providing users with clearer guidance on how much and what type of mHealth intervention use is needed to begin effecting positive behavior and health changes could help encourage sustained use.

Another primary finding was that user engagement was rarely considered a research end point. This is a problem because it is generally expected that users will need to interact with the mHealth technology to experience clinical benefit. Thus, user engagement behavior is a critical aspect of the mHealth intervention itself. Without designating user engagement as a research end point, mHealth intervention trials risk lacking the necessary empirical data and results to help end users understand how to optimally use the technology to see maximal clinical benefit. Lacking these data and results may also be a barrier to informing how these digital tools can be incorporated into regular clinical practice and policy [22]. Thus, in addition to carefully measuring the treatment adherence, self-management behavior, and associated health outcomes directly targeted by the mHealth intervention, equal care should be given to evaluating user engagement, such as how user engagement with the technology may change over time and potentially influence outcomes. Shifting user engagement from afterthought data to an actual research end point would likely improve the quality and rigor of mHealth research and help develop mHealth interventions that motivate users to interact with the technology [318], and, ideally, experience greater clinical benefit.

Our exploratory analysis of studies that provided monetary compensation for study participation versus those that did not (or did not report monetary compensation) showed that engagement-level characterizations were similar between the groups. It is possible that the monetary compensation was provided primarily for completing study procedures (eg, completing study surveys) rather than engaging with the mHealth intervention. In the studies of interventions comprising financial incentives for using the technology or meeting mHealth intervention goals (10/292, 3.4%), no clear pattern emerged for user engagement levels, although this likely reflects the small number of studies incorporating this mHealth component. User engagement evaluation methods were used in similar proportions between the groups, suggesting that monetary compensation was not associated with user engagement measurement selection. An important avenue for future mHealth engagement research is to improve the understanding of how monetary compensation is associated with user engagement levels, particularly when compensation is directed toward mHealth intervention use.

### Limitations

This systematic review is among the most comprehensive reviews on this topic, but it has some limitations. First, due to heterogeneity in the study outcomes, we did not conduct a meta-analysis. Thus, this systematic review cannot conclude which user engagement outcome is associated with the most effective interventions. Second, although we found that study authors tended to frame their results and conclusions to suggest that higher user engagement was associated with positive intervention outcomes, this finding should be considered preliminary and hypothesis generating and does not prove that higher engagement leads to better adherence or self-management, particularly given that study authors may have a tendency to report favorable results and frame their findings in a positive light. However, this is an important area to examine in future research to better understand the characteristics of users with higher mHealth engagement as it relates to adherence and self-management outcomes (eg, highly engaged users may represent a patient group with already high adherence and self-management). Third, we did not exclude studies on the basis of their attrition rate; a future direction for improving the science of engagement is to consider how dropout and withdrawal may influence the characterization of user engagement levels. Fourth, a traditional risk-of-bias assessment [319,320] was not conducted due to our interest in user engagement rather than the primary study results targeting adherence and self-management. If user engagement becomes more commonly designated as a research end point, such evaluations of study quality specific to the evaluation of user engagement will be warranted. Fifth, we included a range of medical and mental health conditions but, due to small sample sizes, were unable to conduct subgroup analyses by diagnosis; as the literature on user engagement grows, it may be possible to explore such differences by diagnosis. Sixth, we focused on the behavioral evaluation of user engagement, although this domain likely involves more than behavior alone [15,321], and these other aspects of user engagement may be examined in a future systematic review. Seventh, our interest was in the measurement and evaluation of mHealth user engagement; thus, we did not consider the numerous individual-level factors that could be related to user engagement. Eighth, although study location was not part of the inclusion criteria, nearly half of the reviewed studies (135/292, 46.2%) were conducted in the United States, which may have influenced the types of intervention studies and approaches to evaluating user engagement. Ninth, we conducted our search in specific scientific databases and did not review gray literature or unpublished results; although our search resulted in 292 studies meeting our inclusion criteria, this systematic review may not be generalizable to all mHealth interventions. Finally, it is necessary to select a cutoff date when conducting a systematic review; yet, new studies are constantly being published. An updated review of the mHealth user engagement literature may be warranted in the future. As the science of mHealth user engagement improves, an important next step is to evaluate how factors related to diversity, equity, and inclusion relate to user engagement to promote wider mHealth use and access.

### Recommendations for Future Researchers

Our systematic review on mHealth user engagement highlighted critical gaps in mHealth adherence and self-management literature as well as opportunities to improve research in this important area of digital health. mHealth researchers need to prioritize the evaluation of user engagement during the study planning phases. Strengthening mHealth user engagement methodological rigor would likely lead to higher-quality data and more impactful study results to guide practice and policy and ultimately encourage the uptake of interventions promoting mHealth adherence or self-management. Furthermore, improved user engagement evaluation may help researchers to identify effective strategies for supporting meaningful user engagement and sustained interest to help users fully experience the intended behavioral and health benefits of the mHealth intervention [16]. Such research is critical for building the evidence base needed to integrate mHealth interventions into regular clinical care and practice guidelines.

The following recommendations for studies of mHealth user engagement are offered:

- Use consistent terminology. Reflecting the most common trends in the literature reviewed, we recommend referring to the measurement of behavioral mHealth use, interaction, or engagement as “use” or “engagement.” Greater consistency in terminology could help improve rigor and consistency in the measurement itself and facilitate comparisons between studies as well as the evaluation of user engagement in meta-analyses.
- Select mHealth user engagement metrics that reflect interactions with the intervention’s key technology components hypothesized to facilitate behavior and health changes.
- Provide mHealth users with expectations for how much they should aim to interact with the technology (give technology dosages when possible).
- Set minimum engagement research benchmarks to scientifically denote the hypothesized user engagement level for a participant to be considered adequately, acceptably, or meaningfully engaged in the intervention.
- Characterize user engagement levels as adequate or acceptable with minimum engagement research benchmarks to help address issues with engagement being characterized as “high” or “low” depending on the metric selected and consider whether the engagement is adequate, meaningful, or effective across metrics (eg, a higher SMS text message response rate and comparatively lower app log-in rate may both be acceptable and meaningful levels of engagement).
- Designate mHealth user engagement as a research end point to help improve the quality and rigor of the mHealth research and the data collected from these studies. Focusing research efforts on user engagement could lead to mHealth engagement recommendations that could ultimately lead to greater clinical improvement.

## Appendix

Multimedia Appendix 1 Search strategy.

Multimedia Appendix 2 Included studies and characteristics.

Multimedia Appendix 3 Supplementary tables.

Multimedia Appendix 4 PRISMA Checklist for Systematic Reviews.

## Abbreviations

mHealth: mobile health
PRISMA: Preferred Reporting Items for Systematic Reviews and Meta-Analyses

## Data Availability

Select review materials are available as multimedia appendices in this manuscript. The manual of procedures and the data sets generated and analyzed during this study are available from the corresponding author on reasonable request. The authors did not use generative AI to write any portions of this manuscript.

## References

[1] (2018). Teens, social media and technology 2018. Pew Research Center.

[2] (2024). Mobile fact sheet. Pew Research Center.

[3] Eaton C, Comer M, Pruette C, Psoter K, Riekert K (2020). Text messaging adherence intervention for adolescents and young adults with chronic kidney disease: pilot randomized controlled trial and stakeholder interviews. J Med Internet Res.

[4] Osborn CY, Hirsch A, Sears LE, Heyman M, Raymond J, Huddleston B, Dachis J (2020). One drop app with an activity tracker for adults with type 1 diabetes: randomized controlled trial. JMIR Mhealth Uhealth.

[5] Rowland SP, Fitzgerald JE, Holme T, Powell J, McGregor A (2020). What is the clinical value of mHealth for patients?. NPJ Digit Med.

[6] Krebs P, Duncan DT (2015). Health app use among US mobile phone owners: a national survey. JMIR Mhealth Uhealth.

[7] Arigo D, Jake-Schoffman DE, Wolin K, Beckjord E, Hekler EB, Pagoto SL (2019). The history and future of digital health in the field of behavioral medicine. J Behav Med.

[8] Cao W, Milks MW, Liu X, Gregory ME, Addison D, Zhang P, Li L (2022). mHealth interventions for self-management of hypertension: framework and systematic review on engagement, interactivity, and tailoring. JMIR Mhealth Uhealth.

[9] Yang Y, Boulton E, Todd C (2022). Measurement of adherence to mHealth physical activity interventions and exploration of the factors that affect the adherence: scoping review and proposed framework. J Med Internet Res.

[10] Schwarz A, Winkens LH, de Vet E, Ossendrijver D, Bouwsema K, Simons M (2023). Design features associated with engagement in mobile health physical activity interventions among youth: systematic review of qualitative and quantitative studies. JMIR Mhealth Uhealth.

[11] Lipschitz JM, Van Boxtel R, Torous J, Firth J, Lebovitz JG, Burdick KE, Hogan TP (2022). Digital mental health interventions for depression: scoping review of user engagement. J Med Internet Res.

[12] Molloy A, Anderson PL (2021). Engagement with mobile health interventions for depression: a systematic review. Internet Interv.

[13] Ng MM, Firth J, Minen M, Torous J (2019). User engagement in mental health apps: a review of measurement, reporting, and validity. Psychiatr Serv.

[14] Short CE, DeSmet A, Woods C, Williams SL, Maher C, Middelweerd A, Müller AM, Wark PA, Vandelanotte C, Poppe L, Hingle MD, Crutzen R (2018). Measuring engagement in eHealth and mHealth behavior change interventions: viewpoint of methodologies. J Med Internet Res.

[15] Perski O, Blandford A, West R, Michie S (2017). Conceptualising engagement with digital behaviour change interventions: a systematic review using principles from critical interpretive synthesis. Transl Behav Med.

[16] Cole-Lewis H, Ezeanochie N, Turgiss J (2019). Understanding health behavior technology engagement: pathway to measuring digital behavior change interventions. JMIR Form Res.

[17] Nieuwlaat R, Wilczynski N, Navarro T, Hobson N, Jeffery R, Keepanasseril A, Agoritsas T, Mistry N, Iorio A, Jack S, Sivaramalingam B, Iserman E, Mustafa RA, Jedraszewski D, Cotoi C, Haynes RB (2014). Interventions for enhancing medication adherence. Cochrane Database Syst Rev.

[18] Mellon L, Doyle F, Hickey A, Ward KD, de Freitas DG, McCormick P, O'Connell O, Conlon P (2022). Interventions for increasing immunosuppressant medication adherence in solid organ transplant recipients. Cochrane Database Syst Rev.

[19] Al-Aqeel S, Gershuni O, Al-Sabhan J, Hiligsmann M (2020). Strategies for improving adherence to antiepileptic drug treatment in people with epilepsy. Cochrane Database Syst Rev.

[20] Covidence systematic review software. Covidence.

[21] IBM SPSS statistics for Windows. IBM Corp.

[22] Forbes G, Newton S, Cantalapiedra Calvete C, Birch J, Dodds J, Steed L, Rivas C, Khan K, Röhricht F, Taylor S, Kahan BC, Ball E (2020). MEMPHIS: a smartphone app using psychological approaches for women with chronic pelvic pain presenting to gynaecology clinics: a randomised feasibility trial. BMJ Open.

[23] Pagoto S, Waring ME (2016). A call for a science of engagement: comment on Rus and Cameron. Ann Behav Med.

[24] Sterne JA, Savović J, Page MJ, Elbers RG, Blencowe NS, Boutron I, Cates CJ, Cheng H, Corbett MS, Eldridge SM, Emberson JR, Hernán MA, Hopewell S, Hróbjartsson A, Junqueira DR, Jüni P, Kirkham JJ, Lasserson T, Li T, McAleenan A, Reeves BC, Shepperd S, Shrier I, Stewart LA, Tilling K, White IR, Whiting PF, Higgins JP (2019). RoB 2: a revised tool for assessing risk of bias in randomised trials. BMJ.

[25] Sterne JA, Hernán MA, Reeves BC, Savović J, Berkman ND, Viswanathan M, Henry D, Altman DG, Ansari MT, Boutron I, Carpenter JR, Chan A, Churchill R, Deeks JJ, Hróbjartsson A, Kirkham J, Jüni P, Loke YK, Pigott TD, Ramsay CR, Regidor D, Rothstein HR, Sandhu L, Santaguida PL, Schünemann HJ, Shea B, Shrier I, Tugwell P, Turner L, Valentine JC, Waddington H, Waters E, Wells GA, Whiting PF, Higgins JP (2016). ROBINS-I: a tool for assessing risk of bias in non-randomised studies of interventions. BMJ.

[26] Torous J, Michalak EE, O'Brien HL (2020). Digital health and engagement-looking behind the measures and methods. JAMA Netw Open.

[27] Achtyes ED, Ben-Zeev D, Luo Z, Mayle H, Burke B, Rotondi AJ, Gottlieb JD, Brunette MF, Mueser KT, Gingerich S, Meyer-Kalos PS, Marcy P, Schooler NR, Robinson DG, Kane JM (2019). Off-hours use of a smartphone intervention to extend support for individuals with schizophrenia spectrum disorders recently discharged from a psychiatric hospital. Schizophr Res.

[28] Adams ZW, Sieverdes JC, Brunner-Jackson B, Mueller M, Chandler J, Diaz V, Patel S, Sox LR, Wilder S, Treiber FA (2018). Meditation smartphone application effects on prehypertensive adults' blood pressure: dose-response feasibility trial. Health Psychol.

[29] Agarwal G, Gaber J, Richardson J, Mangin D, Ploeg J, Valaitis R, Reid GJ, Lamarche L, Parascandalo F, Javadi D, O'Reilly D, Dolovich L (2019). Pilot randomized controlled trial of a complex intervention for diabetes self-management supported by volunteers, technology, and interprofessional primary health care teams. Pilot Feasibility Stud.

[30] Agarwal P, Mukerji G, Desveaux L, Ivers NM, Bhattacharyya O, Hensel JM, Shaw J, Bouck Z, Jamieson T, Onabajo N, Cooper M, Marani H, Jeffs L, Bhatia RS (2019). Mobile app for improved self-management of type 2 diabetes: multicenter pragmatic randomized controlled trial. JMIR Mhealth Uhealth.

[31] Agboola S, Jethwani K, Lopez L, Searl M, O'Keefe S, Kvedar J (2016). Text to move: a randomized controlled trial of a text-messaging program to improve physical activity behaviors in patients with type 2 diabetes mellitus. J Med Internet Res.

[32] Aharonovich E, Stohl M, Cannizzaro D, Hasin D (2017). HealthCall delivered via smartphone to reduce co-occurring drug and alcohol use in HIV-infected adults: a randomized pilot trial. J Subst Abuse Treat.

[33] Amorim AB, Pappas E, Simic M, Ferreira ML, Jennings M, Tiedemann A, Carvalho-E-Silva AP, Caputo E, Kongsted A, Ferreira PH (2019). Integrating mobile-health, health coaching, and physical activity to reduce the burden of chronic low back pain trial (IMPACT): a pilot randomised controlled trial. BMC Musculoskelet Disord.

[34] Angellotti E, Wong JB, Pierce A, Hescott B, Pittas AG (2019). Combining wireless technology and behavioral economics to engage patients (WiBEEP) with cardiometabolic disease: a pilot study. Pilot Feasibility Stud.

[35] Anzaldo-Campos MC, Contreras S, Vargas-Ojeda A, Menchaca-Díaz R, Fortmann A, Philis-Tsimikas A (2016). Dulce wireless Tijuana: a randomized control trial evaluating the impact of project dulce and short-term mobile technology on glycemic control in a family medicine clinic in Northern Mexico. Diabetes Technol Ther.

[36] Arean PA, Hallgren KA, Jordan JT, Gazzaley A, Atkins DC, Heagerty PJ, Anguera JA (2016). The use and effectiveness of mobile apps for depression: results from a fully remote clinical trial. J Med Internet Res.

[37] Arnold C, Williams A, Thomas N (2020). Engaging With a web-based psychosocial intervention for psychosis: qualitative study of user experiences. JMIR Ment Health.

[38] Ashford MT, Olander EK, Rowe H, Fisher JR, Ayers S (2018). Feasibility and acceptability of a web-based treatment with telephone support for postpartum women with anxiety: randomized controlled trial. JMIR Ment Health.

[39] Athilingam P, Jenkins B, Johansson M, Labrador M (2017). A mobile health intervention to improve self-care in patients with heart failure: pilot randomized control trial. JMIR Cardio.

[40] Babbage DR, van Kessel K, Drown J, Thomas S, Sezier A, Thomas P, Kersten P (2019). MS energize: field trial of an app for self-management of fatigue for people with multiple sclerosis. Internet Interv.

[41] Bailey JF, Agarwal V, Zheng P, Smuck M, Fredericson M, Kennedy DJ, Krauss J (2020). Digital care for chronic musculoskeletal pain: 10,000 participant longitudinal cohort study. J Med Internet Res.

[42] Batch BC, Spratt SE, Blalock DV, Benditz C, Weiss A, Dolor RJ, Cho AH (2021). General behavioral engagement and changes in clinical and cognitive outcomes of patients with type 2 diabetes using the Time2Focus mobile app for diabetes education: pilot evaluation. J Med Internet Res.

[43] Belanger HG, Toyinbo P, Barrett B, King E, Sayer NA (2022). Concussion coach for postconcussive symptoms: a randomized, controlled trial of a smartphone application with Afghanistan and Iraq war Veterans. Clin Neuropsychol.

[44] Ben-Zeev D, Brian RM, Jonathan G, Razzano L, Pashka N, Carpenter-Song E, Drake RE, Scherer EA (2018). Mobile health (mHealth) versus clinic-based group intervention for people with serious mental illness: a randomized controlled trial. Psychiatr Serv.

[45] Bennell K, Nelligan RK, Schwartz S, Kasza J, Kimp A, Crofts SJ, Hinman RS (2020). Behavior change text messages for home exercise adherence in knee osteoarthritis: randomized trial. J Med Internet Res.

[46] Bentley CL, Otesile O, Bacigalupo R, Elliott J, Noble H, Hawley MS, Williams EA, Cudd P (2016). Feasibility study of portable technology for weight loss and HbA1c control in type 2 diabetes. BMC Med Inform Decis Mak.

[47] Bentley CL, Powell L, Potter S, Parker J, Mountain GA, Bartlett YK, Farwer J, O'Connor C, Burns J, Cresswell RL, Dunn HD, Hawley MS (2020). The use of a smartphone app and an activity tracker to promote physical activity in the management of chronic obstructive pulmonary disease: randomized controlled feasibility study. JMIR Mhealth Uhealth.

[48] Benzo RP, Kramer KM, Hoult JP, Anderson PM, Begue IM, Seifert SJ (2018). Development and feasibility of a home pulmonary rehabilitation program with health coaching. Respir Care.

[49] Berry D, Blonquist T, Nayak M, Grenon N, Momani T, McCleary N (2018). Self-care support for patients with gastrointestinal cancer: iCancerHealth. Appl Clin Inform.

[50] Bhatia A, Kara J, Janmohamed T, Prabhu A, Lebovic G, Katz J, Clarke H (2021). User engagement and clinical impact of the manage my pain app in patients with chronic pain: a real-world, multi-site trial. JMIR Mhealth Uhealth.

[51] Birney AJ, Gunn R, Russell JK, Ary DV (2016). MoodHacker mobile web app with email for adults to self-manage mild-to-moderate depression: randomized controlled trial. JMIR Mhealth Uhealth.

[52] Blonigen DM, Harris-Olenak B, Kuhn E, Timko C, Humphreys K, Smith JS, Dulin P (2021). Using peers to increase veterans' engagement in a smartphone application for unhealthy alcohol use: a pilot study of acceptability and utility. Psychol Addict Behav.

[53] Boal AL, Abroms LC, Simmens S, Graham AL, Carpenter KM (2016). Combined quitline counseling and text messaging for smoking cessation: a quasi-experimental evaluation. Nicotine Tob Res.

[54] Bobrow K, Farmer AJ, Springer D, Shanyinde M, Yu L, Brennan T, Rayner B, Namane M, Steyn K, Tarassenko L, Levitt N (2016). Mobile phone text messages to support treatment adherence in adults with high blood pressure (SMS-Text Adherence Support [StAR]): a single-blind, randomized trial. Circulation.

[55] Boer L, Bischoff E, van der Heijden M, Lucas P, Akkermans R, Vercoulen J, Heijdra Y, Assendelft W, Schermer T (2019). A smart mobile health tool versus a paper action plan to support self-management of chronic obstructive pulmonary disease exacerbations: randomized controlled trial. JMIR Mhealth Uhealth.

[56] Boettcher J, Magnusson K, Marklund A, Berglund E, Blomdahl R, Braun U, Delin L, Lundén C, Sjöblom K, Sommer D, von Weber K, Andersson G, Carlbring P (2018). Adding a smartphone app to internet-based self-help for social anxiety: a randomized controlled trial. Comput Human Behav.

[57] Boisseau CL, Schwartzman CM, Lawton J, Mancebo MC (2017). App-guided exposure and response prevention for obsessive compulsive disorder: an open pilot trial. Cogn Behav Ther.

[58] Bonar EE, Cunningham RM, Sweezea EC, Blow FC, Drislane LE, Walton MA (2021). Piloting a brief intervention plus mobile boosters for drug use among emerging adults receiving emergency department care. Drug Alcohol Depend.

[59] Bonato M, Turrini F, De Zan V, Meloni A, Plebani M, Brambilla E, Giordani A, Vitobello C, Caccia R, Piacentini MF, LA Torre A, Lazzarin A, Merati G, Galli L, Cinque P (2020). A mobile application for exercise intervention in people living with HIV. Med Sci Sports Exerc.

[60] Boon M, Calvo-Lerma J, Claes I, Havermans T, Asseiceira I, Bulfamante A, Garriga M, Masip E, van Schijndel B, Fornes V, Barreto C, Colombo C, Crespo P, Vicente S, Janssens H, Hulst J, Witters P, Nobili R, Pereira L, Ruperto M, Van der Wiel E, Mainz J, De Boeck K, Ribes-Koninckx C (2020). Use of a mobile application for self-management of pancreatic enzyme replacement therapy is associated with improved gastro-intestinal related quality of life in children with cystic fibrosis. J Cyst Fibros.

[61] Børøsund E, Varsi C, Clark MM, Ehlers SL, Andrykowski MA, Sleveland HR, Bergland A, Nes LS (2020). Pilot testing an app-based stress management intervention for cancer survivors. Transl Behav Med.

[62] Bradway M, Pfuhl G, Joakimsen R, Ribu L, Grøttland A, Årsand E (2018). Analysing mHealth usage logs in RCTs: explaining participants' interactions with type 2 diabetes self-management tools. PLoS One.

[63] Bricker JB, Copeland W, Mull KE, Zeng EY, Watson NL, Akioka KJ, Heffner JL (2017). Drug Alcohol Depend.

[64] Bricker JB, Levin M, Lappalainen R, Mull K, Sullivan B, Santiago-Torres M (2021). Mechanisms of smartphone apps for cigarette smoking cessation: results of a serial mediation model from the iCanQuit randomized trial. JMIR Mhealth Uhealth.

[65] Bricker JB, Watson NL, Mull KE, Sullivan BM, Heffner JL (2020). Efficacy of smartphone applications for smoking cessation: a randomized clinical trial. JAMA Intern Med.

[66] Britto MT, Rohan JM, Dodds CM, Byczkowski TL (2017). A randomized trial of user-controlled text messaging to improve asthma outcomes: a pilot study. Clin Pediatr (Phila).

[67] Browne S, Kechadi MT, O'Donnell S, Dow M, Tully L, Doyle G, O'Malley G (2020). Mobile health apps in pediatric obesity treatment: process outcomes from a feasibility study of a multicomponent intervention. JMIR Mhealth Uhealth.

[68] Bucci S, Barrowclough C, Ainsworth J, Machin M, Morris R, Berry K, Emsley R, Lewis S, Edge D, Buchan I, Haddock G (2018). Actissist: proof-of-concept trial of a theory-driven digital intervention for psychosis. Schizophr Bull.

[69] Bughin F, Bui G, Ayoub B, Blervaque L, Saey D, Avignon A, Brun JF, Molinari N, Pomies P, Mercier J, Gouzi F, Hayot M (2021). Impact of a mobile telerehabilitation solution on metabolic health outcomes and rehabilitation adherence in patients with obesity: randomized controlled trial. JMIR Mhealth Uhealth.

[70] Buis LR, Roberson DN, Kadri R, Rockey NG, Plegue MA, Danak SU, Guetterman TC, Johnson MG, Choe HM, Richardson CR (2020). Understanding the feasibility, acceptability, and efficacy of a clinical pharmacist-led mobile approach (BPTrack) to hypertension management: mixed methods pilot study. J Med Internet Res.

[71] Buscemi J, Buitrago D, Iacobelli F, Penedo F, Maciel C, Guitleman J, Balakrishnan A, Corden M, Adler RF, Bouchard LC, Perez-Tamayo A, Yanez BR (2019). Feasibility of a smartphone-based pilot intervention for Hispanic breast cancer survivors: a brief report. Transl Behav Med.

[72] Butryn ML, Martinelli MK, Crane NT, Godfrey K, Roberts SR, Zhang F, Forman EM (2020). Counselor surveillance of digital self-monitoring data: a pilot randomized controlled trial. Obesity (Silver Spring).

[73] Canan CE, Waselewski ME, Waldman AL, Reynolds G, Flickinger TE, Cohn WF, Ingersoll K, Dillingham R (2020). Long term impact of PositiveLinks: clinic-deployed mobile technology to improve engagement with HIV care. PLoS One.

[74] Cartujano-Barrera F, Peña-Vargas CI, Arana-Chicas E, Pérez-Ramos JG, Mattei J, Hurtado-de-Mendoza A, Costas-Muñiz R, Jiménez J, Cupertino A, Castro E (2021). Decídetexto: feasibility and acceptability of a mobile smoking cessation intervention in Puerto Rico. Int J Environ Res Public Health.

[75] Cartujano-Barrera F, Rodríguez-Bolaños R, Arana-Chicas E, Gallegos-Carrillo K, N Flores Y, Pérez-Rubio G, Falfán-Valencia R, F Ellerbeck E, Reynales-Shigematsu LM, Cupertino AP (2020). Enhancing nicotine replacement therapy usage and adherence through a mobile intervention: secondary data analysis of a single-arm feasibility study in Mexico. Tob Induc Dis.

[76] Castensøe-Seidenfaden P, Husted GR, Jensen AK, Hommel E, Olsen B, Pedersen-Bjergaard U, Kensing F, Teilmann G (2018). Testing a smartphone app (young with diabetes) to improve self-management of diabetes over 12 months: randomized controlled trial. JMIR Mhealth Uhealth.

[77] Cedars A, Blackmore C (2019). Use of a disease-specific mobile health application in the care of adults with congenital heart disease. Proc (Bayl Univ Med Cent).

[78] Chavez K, Palfai TP (2020). Feasibility of a mobile messaging-enhanced brief intervention for high risk heavy drinking MSM: a pre-pilot study. Alcohol Treat Q.

[79] Chen J, Kaye L, Tuffli M, Barrett MA, Jones-Ford S, Shenouda T, Gondalia R, Henderson K, Combs V, Van Sickle D, Stempel DA (2019). Passive monitoring of short-acting beta-agonist use via digital platform in patients with chronic obstructive pulmonary disease: quality improvement retrospective analysis. JMIR Form Res.

[80] Cho H, Flynn G, Saylor M, Gradilla M, Schnall R (2019). Use of the FITT framework to understand patients' experiences using a real-time medication monitoring pill bottle linked to a mobile-based HIV self-management app: a qualitative study. Int J Med Inform.

[81] Cho H, Porras T, Baik D, Beauchemin M, Schnall R (2018). Understanding the predisposing, enabling, and reinforcing factors influencing the use of a mobile-based HIV management app: a real-world usability evaluation. Int J Med Inform.

[82] Choi SA, Lim K, Baek H, Yoo S, Cho A, Kim H, Hwang H, Kim KJ (2021). Impact of mobile health application on data collection and self-management of epilepsy. Epilepsy Behav.

[83] Clements MA, Staggs VS (2017). A mobile app for synchronizing glucometer data: impact on adherence and glycemic control among youths with type 1 diabetes in routine care. J Diabetes Sci Technol.

[84] Coorey G, Peiris D, Scaria A, Mulley J, Neubeck L, Hafiz N, Redfern J (2021). An internet-based intervention for cardiovascular disease management integrated with primary care electronic health records: mixed methods evaluation of implementation fidelity and user engagement. J Med Internet Res.

[85] Côté José, Rouleau G, Ramirez-Garcia MP, Auger P, Thomas R, Leblanc J (2020). Effectiveness of a web-based intervention to support medication adherence among people living with HIV: web-based randomized controlled trial. JMIR Public Health Surveill.

[86] Coughlin LN, Nahum-Shani I, Philyaw-Kotov ML, Bonar EE, Rabbi M, Klasnja P, Murphy S, Walton MA (2021). Developing an adaptive mobile intervention to address risky substance use among adolescents and emerging adults: usability study. JMIR Mhealth Uhealth.

[87] Crafoord M, Fjell M, Sundberg K, Nilsson M, Langius-Eklöf A (2020). Engagement in an interactive app for symptom self-management during treatment in patients with breast or prostate cancer: mixed methods study. J Med Internet Res.

[88] Crane D, Garnett C, Michie S, West R, Brown J (2018). A smartphone app to reduce excessive alcohol consumption: identifying the effectiveness of intervention components in a factorial randomised control trial. Sci Rep.

[89] Creary S, Chisolm D, Stanek J, Hankins J, O'Brien SH (2019). A multidimensional electronic hydroxyurea adherence intervention for children with sickle cell disease: single-arm before-after study. JMIR Mhealth Uhealth.

[90] Dahne J, Collado A, Lejuez CW, Risco CM, Diaz VA, Coles L, Kustanowitz J, Zvolensky MJ, Carpenter MJ (2019). Pilot randomized controlled trial of a Spanish-language behavioral activation mobile app (¡Aptívate!) for the treatment of depressive symptoms among united states Latinx adults with limited English proficiency. J Affect Disord.

[91] Dahne J, Lejuez C, Diaz VA, Player MS, Kustanowitz J, Felton JW, Carpenter MJ (2019). Pilot randomized trial of a self-help behavioral activation mobile app for utilization in primary care. Behav Ther.

[92] Davies EH, Fieggen K, Wilmshurst J, Anyanwu O, Burman RJ, Komarzynski S (2021). Demonstrating the feasibility of digital health to support pediatric patients in South Africa. Epilepsia Open.

[93] Davis SR, Peters D, Calvo RA, Sawyer SM, Foster JM, Smith LD (2021). A consumer designed smartphone app for young people with asthma: pilot of engagement and acceptability. J Asthma.

[94] Davoudi A, Lee NS, Chivers C, Delaney T, Asch EL, Reitz C, Mehta SJ, Chaiyachati KH, Mowery DL (2020). Patient interaction phenotypes with an automated remote hypertension monitoring program and their association with blood pressure control: observational study. J Med Internet Res.

[95] Dawson J, Campbell KL, Craig JC, Tong A, Teixeira-Pinto A, Brown MA, Howard K, Howell M, Khalid R, Sud K, Thiagalingam A, Chow CK, Lee VW (2021). A text messaging intervention for dietary behaviors for people receiving maintenance hemodialysis: a feasibility study of KIDNEYTEXT. Am J Kidney Dis.

[96] de Jong M, van der Meulen-de Jong A, Romberg-Camps M, Degens J, Becx M, Markus T, Tomlow H, Cilissen M, Ipenburg N, Verwey M, Colautti-Duijsens L, Hameeteman W, Masclee A, Jonkers D, Pierik M (2017). Development and feasibility study of a telemedicine tool for all patients with IBD: MyIBDcoach. Inflamm Bowel Dis.

[97] de la Vega R, Ritterband L, Palermo TM (2020). Assessing digital health implementation for a pediatric chronic pain intervention: comparing the RE-AIM and BIT frameworks against real-world trial data and recommendations for future studies. J Med Internet Res.

[98] DeFulio A, Devoto A, Traxler H, Cosottile D, Fingerhood M, Nuzzo P, Dallery J (2021). Smartphone-based incentives for promoting adherence to antiretroviral therapy: a randomized controlled trial. Prev Med Rep.

[99] Desteghe L, Kluts K, Vijgen J, Koopman P, Dilling-Boer D, Schurmans J, Dendale P, Heidbuchel H (2017). The health buddies app as a novel tool to improve adherence and knowledge in atrial fibrillation patients: a pilot study. JMIR Mhealth Uhealth.

[100] Desveaux L, Shaw J, Saragosa M, Soobiah C, Marani H, Hensel J, Agarwal P, Onabajo N, Bhatia RS, Jeffs L (2018). A mobile app to improve self-management of individuals with type 2 diabetes: qualitative realist evaluation. J Med Internet Res.

[101] Dietrich JE, Yee DL, Santos XM, Bercaw-Pratt JL, Kurkowski J, Soni H, Lee-Kim YJ, Shah MD, Mahoney D, Srivaths LV (2017). Assessment of an electronic intervention in young women with heavy menstrual bleeding. J Pediatr Adolesc Gynecol.

[102] Dillingham R, Ingersoll K, Flickinger TE, Waldman AL, Grabowski M, Laurence C, Wispelwey E, Reynolds G, Conaway M, Cohn WF (2018). PositiveLinks: a mobile health intervention for retention in HIV care and clinical outcomes with 12-month follow-up. AIDS Patient Care STDS.

[103] Dombrowski SU, McDonald M, van der Pol M, Grindle M, Avenell A, Carroll P, Calveley E, Elders A, Glennie N, Gray CM, Harris FM, Hapca A, Jones C, Kee F, McKinley MC, Skinner R, Tod M, Hoddinott P (2020). Game of Stones: feasibility randomised controlled trial of how to engage men with obesity in text message and incentive interventions for weight loss. BMJ Open.

[104] Downs DS, Savage JS, Rivera DE, Pauley AM, Leonard KS, Hohman EE, Guo P, McNitt KM, Stetter C, Kunselman A (2021). Adaptive, behavioral intervention impact on weight gain, physical activity, energy intake, and motivational determinants: results of a feasibility trial in pregnant women with overweight/obesity. J Behav Med.

[105] Dunn CG, Turner-McGrievy GM, Wilcox S, Hutto B (2019). Dietary self-monitoring through calorie tracking but not through a digital photography app is associated with significant weight loss: the 2SMART pilot study-a 6-month randomized trial. J Acad Nutr Diet.

[106] Dyal N, McAssey K, Agarwal G (2017). Evaluation of a computerized self-management tool for children with type 1 diabetes: a pilot project. Can J Diabetes.

[107] Ellis TD, Cavanaugh JT, DeAngelis T, Hendron K, Thomas CA, Saint-Hilaire M, Pencina K, Latham NK (2019). Comparative effectiveness of mHealth-supported exercise compared with exercise alone for people with Parkinson disease: randomized controlled pilot study. Phys Ther.

[108] Escobar-Viera C, Zhou Z, Morano JP, Lucero R, Lieb S, McIntosh S, Clauson KA, Cook RL (2020). The Florida mobile health adherence project for people living with HIV (FL-mAPP): longitudinal assessment of feasibility, acceptability, and clinical outcomes. JMIR Mhealth Uhealth.

[109] Fedele DA, Thomas JG, McConville A, McQuaid EL, Voorhees S, Janicke DM, Abu-Hasan M, Chi X, Gurka MJ (2021). Using mobile health to improve asthma self-management in early adolescence: a pilot randomized controlled trial. J Adolesc Health.

[110] Ferrante JM, Devine KA, Bator A, Rodgers A, Ohman-Strickland PA, Bandera EV, Hwang KO (2020). Feasibility and potential efficacy of commercial mHealth/eHealth tools for weight loss in African American breast cancer survivors: pilot randomized controlled trial. Transl Behav Med.

[111] Fogarty AS, Proudfoot J, Whittle EL, Clarke J, Player MJ, Christensen H, Wilhelm K (2017). Preliminary evaluation of a brief web and mobile phone intervention for men with depression: men's positive coping strategies and associated depression, resilience, and work and social functioning. JMIR Ment Health.

[112] Forman EM, Goldstein SP, Crochiere RJ, Butryn ML, Juarascio AS, Zhang F, Foster GD (2019). Randomized controlled trial of OnTrack, a just-in-time adaptive intervention designed to enhance weight loss. Transl Behav Med.

[113] Garofalo R, Kuhns LM, Hotton A, Johnson A, Muldoon A, Rice D (2016). A randomized controlled trial of personalized text message reminders to promote medication adherence among HIV-positive adolescents and young adults. AIDS Behav.

[114] Gazit T, Gutman M, Beatty AL (2021). Assessment of hypertension control among adults participating in a mobile technology blood pressure self-management program. JAMA Netw Open.

[115] Gentili C, Zetterqvist V, Rickardsson J, Holmström L, Simons LE, Wicksell RK (2021). ACTsmart: guided smartphone-delivered acceptance and commitment therapy for chronic pain-a pilot trial. Pain Med.

[116] Gimbel RW, Rennert LM, Crawford P, Little JR, Truong K, Williams JE, Griffin SF, Shi L, Chen L, Zhang L, Moss JB, Marshall RC, Edwards KW, Crawford KJ, Hing M, Schmeltz A, Lumsden B, Ashby M, Haas E, Palazzo K (2020). Enhancing patient activation and self-management activities in patients with type 2 diabetes using the US Department of Defense mobile health care environment: feasibility study. J Med Internet Res.

[117] Gire N, Caton N, McKeown M, Mohmed N, Duxbury J, Kelly J, Riley M, J Taylor P, Taylor CD, Naeem F, Chaudhry IB, Husain N (2021). Care co-ordinator in my pocket': a feasibility study of mobile assessment and therapy for psychosis (TechCare). BMJ Open.

[118] Godersky ME, Klein JW, Merrill JO, Blalock KL, Saxon AJ, Samet JH, Tsui JI (2020). Acceptability and feasibility of a mobile health application for video directly observed therapy of buprenorphine for opioid use disorders in an office-based setting. J Addict Med.

[119] Godino JG, Golaszewski NM, Norman GJ, Rock CL, Griswold WG, Arredondo E, Marshall S, Kolodziejczyk J, Dillon L, Raab F, Jain S, Crawford M, Merchant G, Patrick K (2019). Text messaging and brief phone calls for weight loss in overweight and obese English- and Spanish-speaking adults: a 1-year, parallel-group, randomized controlled trial. PLoS Med.

[120] Gordon JS, Armin J, D Hingle M, Giacobbi P, Cunningham JK, Johnson T, Abbate K, Howe CL, Roe DJ (2017). Development and evaluation of the See Me Smoke-Free multi-behavioral mHealth app for women smokers. Transl Behav Med.

[121] Goyal S, Nunn CA, Rotondi M, Couperthwaite AB, Reiser S, Simone A, Katzman DK, Cafazzo JA, Palmert MR (2017). A mobile app for the self-management of type 1 diabetes among adolescents: a randomized controlled trial. JMIR Mhealth Uhealth.

[122] Greer JA, Jacobs JM, Pensak N, Nisotel LE, Fishbein JN, MacDonald JJ, Ream ME, Walsh EA, Buzaglo J, Muzikansky A, Lennes IT, Safren SA, Pirl WF, Temel JS (2020). Randomized trial of a smartphone mobile app to improve symptoms and adherence to oral therapy for cancer. J Natl Compr Canc Netw.

[123] Guarino H, Acosta M, Marsch LA, Xie H, Aponte-Melendez Y (2016). A mixed-methods evaluation of the feasibility, acceptability, and preliminary efficacy of a mobile intervention for methadone maintenance clients. Psychol Addict Behav.

[124] Guo X, Gu X, Jiang J, Li H, Duan R, Zhang Y, Sun L, Bao Z, Shen J, Chen F (2019). A hospital-community-family-based telehealth program for patients with chronic heart failure: single-arm, prospective feasibility study. JMIR Mhealth Uhealth.

[125] Guthrie NL, Berman MA, Edwards KL, Appelbaum KJ, Dey S, Carpenter J, Eisenberg DM, Katz DL (2019). Achieving rapid blood pressure control with digital therapeutics: retrospective cohort and machine learning study. JMIR Cardio.

[126] Haas K, Martin A, Park KT (2017). Text message intervention (TEACH) improves quality of life and patient activation in celiac disease: a randomized clinical trial. J Pediatr.

[127] Hammond AS, Sweeney MM, Chikosi TU, Stitzer ML (2021). Digital delivery of a contingency management intervention for substance use disorder: a feasibility study with DynamiCare Health. J Subst Abuse Treat.

[128] Han A, Min SI, Ahn S, Min S, Hong H, Han N, Kim YS, Ahn C, Ha J (2019). Mobile medication manager application to improve adherence with immunosuppressive therapy in renal transplant recipients: a randomized controlled trial. PLoS One.

[129] Hardcastle SJ, Jiménez-Castuera R, Maxwell-Smith C, Bulsara MK, Hince D (2020). Fitbit wear-time and patterns of activity in cancer survivors throughout a physical activity intervention and follow-up: exploratory analysis from a randomised controlled trial. PLoS One.

[130] Harzand A, Witbrodt B, Davis-Watts ML, Alrohaibani A, Goese D, Wenger NK, Shah AJ, Zafari AM (2018). Feasibility of a smartphone-enabled cardiac rehabilitation program in male veterans with previous clinical evidence of coronary heart disease. Am J Cardiol.

[131] Heale LD, Dover S, Goh YI, Maksymiuk VA, Wells GD, Feldman BM (2018). A wearable activity tracker intervention for promoting physical activity in adolescents with juvenile idiopathic arthritis: a pilot study. Pediatr Rheumatol Online J.

[132] Hébert ET, Ra CK, Alexander AC, Helt A, Moisiuc R, Kendzor DE, Vidrine DJ, Funk-Lawler RK, Businelle MS (2020). A mobile just-in-time adaptive intervention for smoking cessation: pilot randomized controlled trial. J Med Internet Res.

[133] Heminger CL, Boal AL, Zumer M, Abroms LC (2016). Text2Quit: an analysis of participant engagement in the mobile smoking cessation program. Am J Drug Alcohol Abuse.

[134] Herbec A, Brown J, Shahab L, West R, Raupach T (2019). Pragmatic randomised trial of a smartphone app (NRT2Quit) to improve effectiveness of nicotine replacement therapy in a quit attempt by improving medication adherence: results of a prematurely terminated study. Trials.

[135] Herbert LJ, Collier S, Stern A, Monaghan M, Streisand R (2016). A pilot test of the self-management and research technology project: a text message-based diabetes self-management program for adolescents. J Child Health Care.

[136] Hightow-Weidman L, Muessig KE, Egger JR, Vecchio A, Platt A (2021). Epic Allies: a gamified mobile app to improve engagement in HIV care and antiretroviral adherence among young men who have sex with men. AIDS Behav.

[137] Hightow-Weidman L, Muessig K, Knudtson K, Srivatsa M, Lawrence E, LeGrand S, Hotten A, Hosek S (2018). A gamified smartphone app to support engagement in care and medication adherence for HIV-positive young men who have sex with men (AllyQuest): development and pilot study. JMIR Public Health Surveill.

[138] Hilliard ME, Cao VT, Eshtehardi SS, Minard CG, Saber R, Thompson D, Karaviti LP, Anderson BJ (2020). Type 1 doing well: pilot feasibility and acceptability study of a strengths-based mHealth app for parents of adolescents with type 1 diabetes. Diabetes Technol Ther.

[139] Himelhoch S, Kreyenbuhl J, Palmer-Bacon J, Chu M, Brown C, Potts W (2017). Pilot feasibility study of Heart2HAART: a smartphone application to assist with adherence among substance users living with HIV. AIDS Care.

[140] Hoffman V, Söderström L, Samuelsson E (2017). Self-management of stress urinary incontinence via a mobile app: two-year follow-up of a randomized controlled trial. Acta Obstet Gynecol Scand.

[141] Hommel KA, Carmody J, Hershey AD, Holbein C, Kabbouche-Samaha M, Peugh J, Powers S (2020). Digital therapeutic self-management intervention in adolescents with migraine: feasibility and preliminary efficacy of "migraine manager". Headache.

[142] Hood A, Nwankwo C, Walton A, McTate E, Joffe N, Quinn CT, Britto MT, Peugh J, Mara CA, Crosby LE (2021). Mobile health use predicts self-efficacy and self-management in adolescents with sickle cell disease. Transl Behav Med.

[143] Horner GN, Agboola S, Jethwani K, Tan-McGrory A, Lopez L (2017). Designing patient-centered text messaging interventions for increasing physical activity among participants with type 2 diabetes: qualitative results from the text to move intervention. JMIR Mhealth Uhealth.

[144] Horvath KJ, Lammert S, MacLehose RF, Danh T, Baker JV, Carrico AW (2019). A pilot study of a mobile app to support HIV antiretroviral therapy adherence among men who have sex with men who use stimulants. AIDS Behav.

[145] Huang Z, Tan E, Lum E, Sloot P, Boehm BO, Car J (2019). A smartphone app to improve medication adherence in patients with type 2 diabetes in Asia: feasibility randomized controlled trial. JMIR Mhealth Uhealth.

[146] Huberty JL, Green J, Puzia ME, Larkey L, Laird B, Vranceanu A, Vlisides-Henry R, Irwin MR (2021). Testing a mindfulness meditation mobile app for the treatment of sleep-related symptoms in adults with sleep disturbance: a randomized controlled trial. PLoS One.

[147] Huh U, Tak YJ, Song S, Chung SW, Sung SM, Lee CW, Bae M, Ahn HY (2019). Feedback on physical activity through a wearable device connected to a mobile phone app in patients with metabolic syndrome: pilot study. JMIR Mhealth Uhealth.

[148] Hunt M, Miguez S, Dukas B, Onwude O, White S (2021). Efficacy of Zemedy, a mobile digital therapeutic for the self-management of irritable bowel syndrome: crossover randomized controlled trial. JMIR Mhealth Uhealth.

[149] Huo X, Krumholz HM, Bai X, Spatz ES, Ding Q, Horak P, Zhao W, Gong Q, Zhang H, Yan X, Sun Y, Liu J, Wu X, Guan W, Wang X, Li J, Li X, Spertus JA, Masoudi FA, Zheng X (2019). Effects of mobile text messaging on glycemic control in patients with coronary heart disease and diabetes mellitus: a randomized clinical trial. Circ Cardiovasc Qual Outcomes.

[150] Hutchesson MJ, Tan CY, Morgan P, Callister R, Collins C (2016). Enhancement of self-monitoring in a web-based weight loss program by extra individualized feedback and reminders: randomized trial. J Med Internet Res.

[151] Irizarry T, Allen M, Suffoletto BP, Einhorn J, Burke LE, Kamarck TW, Rollman BL, Muldoon MF (2018). Development and preliminary feasibility of an automated hypertension self-management system. Am J Med.

[152] Ivanova E, Lindner P, Ly KH, Dahlin M, Vernmark K, Andersson G, Carlbring P (2016). Guided and unguided acceptance and commitment therapy for social anxiety disorder and/or panic disorder provided via the internet and a smartphone application: a randomized controlled trial. J Anxiety Disord.

[153] Jácome C, Almeida R, Pereira AM, Amaral R, Mendes S, Alves-Correia M, Vidal C, López Freire S, Méndez Brea P, Araújo L, Couto M, Antolín-Amérigo D, de la Hoz Caballer B, Barra Castro A, Gonzalez-De-Olano D, Todo Bom A, Azevedo J, Leiria Pinto P, Pinto N, Castro Neves A, Palhinha A, Todo Bom F, Costa A, Chaves Loureiro C, Maia Santos L, Arrobas A, Valério M, Cardoso J, Emiliano M, Gerardo R, Cidrais Rodrigues JC, Oliveira G, Carvalho J, Mendes A, Lozoya C, Santos N, Menezes F, Gomes R, Câmara R, Rodrigues Alves R, Moreira AS, Bordalo D, Alves C, Ferreira JA, Lopes C, Silva D, Vasconcelos MJ, Teixeira MF, Ferreira-Magalhães M, Taborda-Barata L, Cálix MJ, Alves A, Almeida Fonseca J (2021). Feasibility and acceptability of an asthma app to monitor medication adherence: mixed methods study. JMIR Mhealth Uhealth.

[154] Jaimini U, Thirunarayan K, Kalra M, Venkataraman R, Kadariya D, Sheth A (2018). "How is my child's asthma?" Digital phenotype and actionable insights for pediatric asthma. JMIR Pediatr Parent.

[155] Jamison RN, Mei A, Ross EL (2018). Longitudinal trial of a smartphone pain application for chronic pain patients: Predictors of compliance and satisfaction. J Telemed Telecare.

[156] Johansson M, Berman AH, Sinadinovic K, Lindner P, Hermansson U, Andréasson S (2021). Effects of internet-based cognitive behavioral therapy for harmful alcohol use and alcohol dependence as self-help or with therapist guidance: three-armed randomized trial. J Med Internet Res.

[157] Jonassaint CR, Kang C, Prussien KV, Yarboi J, Sanger MS, Wilson JD, De Castro L, Shah N, Sarkar U (2020). Feasibility of implementing mobile technology-delivered mental health treatment in routine adult sickle cell disease care. Transl Behav Med.

[158] Jonathan G, Carpenter-Song EA, Brian RM, Ben-Zeev D (2019). Life with FOCUS: a qualitative evaluation of the impact of a smartphone intervention on people with serious mental illness. Psychiatr Rehabil J.

[159] Juarascio AS, Hunt RA, Lantz Lesser E, Engel SG, Pisetsky EM, Peterson CB, Wonderlich SA (2021). Enhancing integrative cognitive-affective therapy with ecological momentary interventions: a pilot trial. Eur Eat Disord Rev.

[160] Kählke F, Berger T, Schulz A, Baumeister H, Berking M, Auerbach RP, Bruffaerts R, Cuijpers P, Kessler RC, Ebert DD (2019). Efficacy of an unguided internet-based self-help intervention for social anxiety disorder in university students: a randomized controlled trial. Int J Methods Psychiatr Res.

[161] Kanera IM, Bolman CA, Willems RA, Mesters I, Lechner L (2016). Lifestyle-related effects of the web-based Kanker Nazorg Wijzer (Cancer Aftercare Guide) intervention for cancer survivors: a randomized controlled trial. J Cancer Surviv.

[162] Kanera IM, Willems RA, Bolman CA, Mesters I, Zambon V, Gijsen BC, Lechner L (2016). Use and appreciation of a tailored self-management eHealth intervention for early cancer survivors: process evaluation of a randomized controlled trial. J Med Internet Res.

[163] Kanera IM, Willems RA, Bolman CA, Mesters I, Verboon P, Lechner L (2017). Long-term effects of a web-based cancer aftercare intervention on moderate physical activity and vegetable consumption among early cancer survivors: a randomized controlled trial. Int J Behav Nutr Phys Act.

[164] Kassavou A, A'Court CE, Chauhan J, Brimocombe JD, Bhattacharya D, Naughton F, Hardeman W, Mascolo C, Sutton S (2020). Assessing the acceptability of a text messaging service and smartphone app to support patient adherence to medications prescribed for high blood pressure: a pilot study. Pilot Feasibility Stud.

[165] Kaushal T, Katz LE, Joseph J, Marowitz M, Morales KH, Atkins D, Ritter D, Simon R, Laffel L, Lipman TH (2022). A text messaging intervention with financial incentive for adolescents with type 1 diabetes. J Diabetes Sci Technol.

[166] Kauw D, Koole MA, Winter MM, Dohmen DA, Tulevski II, Blok S, Somsen GA, Schijven MP, Vriend JW, Robbers-Visser D, Mulder BJ, Bouma BJ, Schuuring MJ (2019). Advantages of mobile health in the management of adult patients with congenital heart disease. Int J Med Inform.

[167] Kay MC, Burroughs J, Askew S, Bennett GG, Armstrong S, Steinberg DM (2018). Digital weight loss intervention for parents of children being treated for obesity: a prospective cohort feasibility trial. J Med Internet Res.

[168] Kelechi TJ, Prentice MA, Mueller M, Madisetti M, Vertegel A (2020). A lower leg physical activity intervention for individuals with chronic venous leg ulcers: randomized controlled trial. JMIR Mhealth Uhealth.

[169] Kenyon CC, Sundar KG, Gruschow SM, Quarshie WO, Feudtner C, Bryant-Stephens TC, Miller VA (2020). Tailored medication adherence incentives for high-risk children with asthma: a pilot study. J Asthma.

[170] Kim H, Faw M, Michaelides A (2017). Mobile but connected: harnessing the power of self-efficacy and group support for weight loss success through mHealth intervention. J Health Commun.

[171] Kim MY, Lee SY, Jo EJ, Lee S, Kang M, Song W, Kim S, Cho S, Min K, Ahn K, Chang Y (2016). Feasibility of a smartphone application based action plan and monitoring in asthma. Asia Pac Allergy.

[172] King-Dowling S, Psihogios AM, Hill-Kayser C, Szalda D, O'Hagan B, Darabos K, Daniel LC, Barakat LP, Fleisher L, Maurer LA, Velázquez-Martin B, Jacobs LA, Hobbie W, Ginsberg JP, Vachani CC, Metz JM, Schwartz LA (2021). Acceptability and feasibility of survivorship care plans and an accompanying mobile health intervention for adolescent and young adult survivors of childhood cancer. Pediatr Blood Cancer.

[173] Klausen SH, Andersen LL, Søndergaard L, Jakobsen JC, Zoffmann V, Dideriksen K, Kruse A, Mikkelsen UR, Wetterslev J (2016). Effects of eHealth physical activity encouragement in adolescents with complex congenital heart disease: the PReVaiL randomized clinical trial. Int J Cardiol.

[174] Kliemann N, Croker H, Johnson F, Beeken RJ (2019). Development of the top tips habit-based weight loss app and preliminary indications of its usage, effectiveness, and acceptability: mixed-methods pilot study. JMIR Mhealth Uhealth.

[175] Kooij L, Vos PJ, Dijkstra A, van Harten WH (2021). Effectiveness of a mobile health and self-management app for high-risk patients with chronic obstructive pulmonary disease in daily clinical practice: mixed methods evaluation study. JMIR Mhealth Uhealth.

[176] Koot D, Goh PS, Lim RS, Tian Y, Yau TY, Tan NC, Finkelstein EA (2019). A mobile lifestyle management program (GlycoLeap) for people with type 2 diabetes: single-arm feasibility study. JMIR Mhealth Uhealth.

[177] Korus M, Cruchley E, Calic M, Gold A, Anthony SJ, Parekh RS, Stinson JN (2020). Assessing the acceptability and efficacy of teens taking charge: transplant-a pilot randomized control trial. Pediatr Transplant.

[178] Kosse RC, Bouvy ML, Belitser SV, de Vries TW, van der Wal PS, Koster ES (2019). Effective engagement of adolescent asthma patients with mobile health-supporting medication adherence. JMIR Mhealth Uhealth.

[179] Krishnakumar A, Verma R, Chawla R, Sosale A, Saboo B, Joshi S, Shaikh M, Shah A, Kolwankar S, Mattoo V (2021). Evaluating glycemic control in patients of South Asian origin with type 2 diabetes using a digital therapeutic platform: analysis of real-world data. J Med Internet Res.

[180] Kubo A, Aghaee S, Kurtovich EM, Nkemere L, Quesenberry CP, McGinnis MK, Avalos LA (2021). mHealth mindfulness intervention for women with moderate-to-moderately-severe antenatal depressive symptoms: a pilot study within an integrated health care system. Mindfulness (N Y).

[181] Kumar S, Moseson H, Uppal J, Juusola JL (2018). A diabetes mobile app with in-app coaching from a certified diabetes educator reduces A1C for individuals with type 2 diabetes. Diabetes Educ.

[182] Lakshminarayana R, Wang D, Burn D, Chaudhuri KR, Galtrey C, Guzman NV, Hellman B, Pal S, Stamford J, Steiger M, Stott RW, Teo J, Barker RA, Wang E, Bloem BR, van der Eijk M, Rochester L, Williams A, Ben James (2017). Using a smartphone-based self-management platform to support medication adherence and clinical consultation in Parkinson's disease. NPJ Parkinsons Dis.

[183] Lalloo C, Hundert A, Harris L, Pham Q, Campbell F, Chorney J, Dick B, Simmonds M, Cafazzo J, Stinson J (2019). Capturing daily disease experiences of adolescents with chronic pain: mHealth-mediated symptom tracking. JMIR Mhealth Uhealth.

[184] Lambert SD, Grover S, Laizner AM, McCusker J, Belzile E, Moodie EE, Kayser JW, Lowensteyn I, Vallis M, Walker M, Da Costa D, Pilote L, Ibberson C, Sabetti J, de Raad M (2022). Adaptive web-based stress management programs among adults with a cardiovascular disease: a pilot Sequential Multiple Assignment Randomized Trial (SMART). Patient Educ Couns.

[185] Leach CR, Hudson SV, Diefenbach MA, Wiseman KP, Sanders A, Coa K, Chantaprasopsuk S, Stephens RL, Alfano CM (2022). Cancer health self-efficacy improvement in a randomized controlled trial. Cancer.

[186] Lee H, Uhm KE, Cheong IY, Yoo JS, Chung SH, Park YH, Lee JY, Hwang JH (2018). Patient satisfaction with mobile health (mHealth) application for exercise intervention in breast cancer survivors. J Med Syst.

[187] Lee KW, Kim HB, Lee SH, Ha HK (2019). Changes in weight and health-related behavior using smartphone applications in patients with colorectal polyps. J Nutr Educ Behav.

[188] Lee MK, Lee DY, Ahn HY, Park CY (2021). A novel user utility score for diabetes management using tailored mobile coaching: secondary analysis of a randomized controlled trial. JMIR Mhealth Uhealth.

[189] Lee SE, Park SK, Park YS, Kim KA, Choi HS, Oh SW (2021). Effects of short-term mobile application use on weight reduction for patients with type 2 diabetes. J Obes Metab Syndr.

[190] Lefler LL, Rhoads SJ, Harris M, Funderburg AE, Lubin SA, Martel ID, Faulkner JL, Rooker JL, Bell DK, Marshall H, Beverly CJ (2018). Evaluating the use of mobile health technology in older adults with heart failure: mixed-methods study. JMIR Aging.

[191] Leon N, Namadingo H, Cooper S, Bobrow K, Mwantisi C, Nyasulu M, Sicwebu N, Crampin A, Levitt N, Farmer A (2021). Process evaluation of a brief messaging intervention to improve diabetes treatment adherence in sub-Saharan Africa. BMC Public Health.

[192] Leonard S, Anderson LM, Jonassaint J, Jonassaint C, Shah N (2017). Utilizing a novel mobile health "selfie" application to improve compliance to iron chelation in pediatric patients receiving chronic transfusions. J Pediatr Hematol Oncol.

[193] Levin JB, Sajatovic M, Rahman M, Aebi ME, Tatsuoka C, Depp C, Cushman C, Johnston E, Cassidy KA, Blixen C, Eskew L, Klein PJ, Fuentes-Casiano E, Moore DJ (2019). Outcomes of psychoeducation and a text messaging adherence intervention among individuals with hypertension and bipolar disorder. Psychiatr Serv.

[194] Lewis S, Ainsworth J, Sanders C, Stockton-Powdrell C, Machin M, Whelan P, Hopkins R, He Z, Applegate E, Drake R, Bamford C, Roberts C, Wykes T (2020). Smartphone-enhanced symptom management in psychosis: open, randomized controlled trial. J Med Internet Res.

[195] Loeckx M, Rabinovich RA, Demeyer H, Louvaris Z, Tanner R, Rubio N, Frei A, De Jong C, Gimeno-Santos E, Rodrigues FM, Buttery SC, Hopkinson NS, Büsching G, Strassmann A, Serra I, Vogiatzis I, Garcia-Aymerich J, Polkey MI, Troosters T (2018). Smartphone-based physical activity telecoaching in chronic obstructive pulmonary disease: mixed-methods study on patient experiences and lessons for implementation. JMIR Mhealth Uhealth.

[196] Lopez KE, Salvy SJ, Fink C, Werner J, Wee CP, Hegedus E, Gonzalez J, Fox DS, Vidmar AP (2021). Executive functioning, depressive symptoms, and intervention engagement in a sample of adolescents enrolled in a weight management program. Child Obes.

[197] Malte CA, Dulin PL, Baer JS, Fortney JC, Danner AN, Lott AM, Hawkins EJ (2021). Usability and acceptability of a mobile app for the self-management of alcohol misuse among Veterans (Step Away): pilot cohort study. JMIR Mhealth Uhealth.

[198] Mamykina L, Heitkemper EM, Smaldone AM, Kukafka R, Cole-Lewis H, Davidson PG, Mynatt ED, Tobin JN, Cassells A, Goodman C, Hripcsak G (2016). Structured scaffolding for reflection and problem solving in diabetes self-management: qualitative study of mobile diabetes detective. J Am Med Inform Assoc.

[199] Mantani A, Kato T, Furukawa TA, Horikoshi M, Imai H, Hiroe T, Chino B, Funayama T, Yonemoto N, Zhou Q, Kawanishi N (2017). Smartphone cognitive behavioral therapy as an adjunct to pharmacotherapy for refractory depression: randomized controlled trial. J Med Internet Res.

[200] Mayberry LS, Berg CA, Greevy RA, Nelson LA, Bergner EM, Wallston KA, Harper KJ, Elasy TA (2021). Mixed-methods randomized evaluation of FAMS: a mobile phone-delivered intervention to improve family/friend involvement in adults' type 2 diabetes self-care. Ann Behav Med.

[201] McClure JB, Anderson ML, Bradley K, An LC, Catz SL (2016). Evaluating an adaptive and interactive mHealth smoking cessation and medication adherence program: a randomized pilot feasibility study. JMIR Mhealth Uhealth.

[202] McGill DE, Volkening LK, Butler DA, Wasserman RM, Anderson BJ, Laffel LM (2019). Text-message responsiveness to blood glucose monitoring reminders is associated with HbA benefit in teenagers with type 1 diabetes. Diabet Med.

[203] McGillicuddy JW, Chandler JL, Sox LR, Taber DJ (2020). Exploratory analysis of the impact of an mHealth medication adherence intervention on tacrolimus trough concentration variability: post hoc results of a randomized controlled trial. Ann Pharmacother.

[204] Melilli E, Cestone G, Revuelta I, Meneghini M, Lladó L, Montero N, Manonelles A, Diaz M, Coloma A, Torregrosa V, Baliellas C, Cruzado JM, Diekmann F, Grinyó J, Bestard O (2021). Adoption of a novel smart mobile-health application technology to track chronic immunosuppression adherence in solid organ transplantation: Results of a prospective, observational, multicentre, pilot study. Clin Transplant.

[205] Melissant HC, Verdonck-de Leeuw IM, Lissenberg-Witte BI, Konings IR, Cuijpers P, Van Uden-Kraan CF (2018). 'Oncokompas', a web-based self-management application to support patient activation and optimal supportive care: a feasibility study among breast cancer survivors. Acta Oncol.

[206] Merwin RM, Moskovich AA, Babyak M, Feinglos M, Honeycutt LK, Mooney J, Freeman SP, Batchelder H, Sangvai D (2021). An open trial of app-assisted acceptance and commitment therapy (iACT) for eating disorders in type 1 diabetes. J Eat Disord.

[207] Minen MT, Adhikari S, Padikkala J, Tasneem S, Bagheri A, Goldberg E, Powers S, Lipton RB (2020). Smartphone-delivered progressive muscle relaxation for the treatment of migraine in primary care: a randomized controlled trial. Headache.

[208] Minen MT, Schaubhut KB, Morio K (2020). Smartphone based behavioral therapy for pain in multiple sclerosis (MS) patients: a feasibility acceptability randomized controlled study for the treatment of comorbid migraine and MS pain. Mult Scler Relat Disord.

[209] Moberg C, Niles A, Beermann D (2019). Guided self-help works: randomized waitlist controlled trial of pacifica, a mobile app integrating cognitive behavioral therapy and mindfulness for stress, anxiety, and depression. J Med Internet Res.

[210] Mollard E, Michaud K (2018). A mobile app with optical imaging for the self-management of hand rheumatoid arthritis: pilot study. JMIR Mhealth Uhealth.

[211] Mollerup A, Harboe G, Johansen JD (2016). User evaluation of patient counselling, combining nurse consultation and eHealth in hand eczema. Contact Dermatitis.

[212] Moore DJ, Pasipanodya EC, Umlauf A, Rooney AS, Gouaux B, Depp CA, Atkinson JH, Montoya JL (2018). Individualized texting for adherence building (iTAB) for methamphetamine users living with HIV: a pilot randomized clinical trial. Drug Alcohol Depend.

[213] Morita PP, Yeung MS, Ferrone M, Taite AK, Madeley C, Stevens Lavigne A, To T, Lougheed MD, Gupta S, Day AG, Cafazzo JA, Licskai C (2019). A patient-centered mobile health system that supports asthma self-management (breathe): design, development, and utilization. JMIR Mhealth Uhealth.

[214] Moyano D, Morelli D, Santero M, Belizan M, Irazola V, Beratarrechea A (2019). Perceptions and acceptability of text messaging for diabetes care in primary care in Argentina: exploratory study. JMIR Diabetes.

[215] Mulvaney SA, Vaala S, Hood KK, Lybarger C, Carroll R, Williams L, Schmidt DC, Johnson K, Dietrich MS, Laffel L (2018). Mobile momentary assessment and biobehavioral feedback for adolescents with type 1 diabetes: feasibility and engagement patterns. Diabetes Technol Ther.

[216] Muralidharan S, Ranjani H, Mohan Anjana R, Jena S, Tandon N, Gupta Y, Ambekar S, Koppikar V, Jagannathan N, Allender S, Mohan V (2019). Engagement and weight loss: results from the mobile health and diabetes trial. Diabetes Technol Ther.

[217] Murphy J, McSharry J, Hynes L, Molloy GJ (2021). A smartphone app to support adherence to inhaled corticosteroids in young adults with asthma: multi-methods feasibility study. JMIR Form Res.

[218] Nelson LA, Greevy RA, Spieker A, Wallston KA, Elasy TA, Kripalani S, Gentry C, Bergner EM, LeStourgeon LM, Williamson SE, Mayberry LS (2021). Effects of a tailored text messaging intervention among diverse adults with type 2 diabetes: evidence from the 15-month REACH randomized controlled trial. Diabetes Care.

[219] Nelson LA, Mulvaney SA, Gebretsadik T, Ho Y, Johnson KB, Osborn CY (2016). Disparities in the use of a mHealth medication adherence promotion intervention for low-income adults with type 2 diabetes. J Am Med Inform Assoc.

[220] Nelson LA, Spieker A, Greevy R, LeStourgeon LM, Wallston KA, Mayberry LS (2020). User engagement among diverse adults in a 12-month text message-delivered diabetes support intervention: results from a randomized controlled trial. JMIR Mhealth Uhealth.

[221] Nichols M, Miller S, Treiber F, Ruggiero K, Dawley E, Teufel Ii R (2020). Patient and parent perspectives on improving pediatric asthma self-management through a mobile health intervention: pilot study. JMIR Form Res.

[222] Nilsson A, Sörman K, Klingvall J, Ovelius E, Lundberg J, Hellner C (2019). MyCompass in a Swedish context - lessons learned from the transfer of a self-guided intervention targeting mental health problems. BMC Psychiatry.

[223] Oehler C, Scholze K, Reich H, Sander C, Hegerl U (2021). Intervention use and symptom change with unguided internet-based cognitive behavioral therapy for depression during the COVID-19 pandemic: log data analysis of a convenience sample. JMIR Ment Health.

[224] Olalla J, García de Lomas JM, Márquez E, González FJ, Del Arco A, De La Torre J, Prada JL, Cantudo F, Martín MD, Nieto M, Perez Stachowski J, García-Alegría J (2019). Experience of using an app in HIV patients older than 60 years: pilot program. JMIR Mhealth Uhealth.

[225] Ong SW, Jassal SV, Miller JA, Porter EC, Cafazzo JA, Seto E, Thorpe KE, Logan AG (2016). Integrating a smartphone-based self-management system into usual care of advanced CKD. Clin J Am Soc Nephrol.

[226] Osborn CY, van Ginkel JR, Rodbard D, Heyman M, Marrero DG, Huddleston B, Dachis J (2017). One Drop | Mobile: an evaluation of hemoglobin A1c improvement linked to app engagement. JMIR Diabetes.

[227] Oser M, Wallace ML, Solano F, Szigethy EM (2019). Guided digital cognitive behavioral program for anxiety in primary care: propensity-matched controlled trial. JMIR Ment Health.

[228] Pagan-Ortiz ME, Goulet P, Kogelman L, Levkoff SE, Weitzman PF (2019). Feasibility of a texting intervention to improve medication adherence among older HIV+ African Americans: A mixed-method pilot study. Gerontol Geriatr Med.

[229] Pagoto S, Tulu B, Waring ME, Goetz J, Bibeau J, Divito J, Groshon L, Schroeder M (2021). Slip Buddy app for weight management: randomized feasibility trial of a dietary lapse tracking app. JMIR Mhealth Uhealth.

[230] Palermo TM, de la Vega R, Murray C, Law E, Zhou C (2020). A digital health psychological intervention (WebMAP Mobile) for children and adolescents with chronic pain: results of a hybrid effectiveness-implementation stepped-wedge cluster randomized trial. Pain.

[231] Patel ML, Hopkins CM, Brooks TL, Bennett GG (2019). Comparing self-monitoring strategies for weight loss in a smartphone app: randomized controlled trial. JMIR Mhealth Uhealth.

[232] Perski O, Watson NL, Mull KE, Bricker JB (2021). Identifying content-based engagement patterns in a smoking cessation website and associations with user characteristics and cessation outcomes: a sequence and cluster analysis. Nicotine Tob Res.

[233] Phillips S, Kanter J, Mueller M, Gulledge A, Ruggiero K, Johnson M, Kelechi TJ (2021). Feasibility of an mHealth self-management intervention for children and adolescents with sickle cell disease and their families. Transl Behav Med.

[234] Poort H, Ryan A, MacDougall K, Malinowski P, MacDonald A, Markin Z, Pirl W, Greer J, Fasciano K (2021). Feasibility and acceptability of a mobile phone app intervention for coping with cancer as a young adult: pilot trial and thematic analysis. J Med Internet Res.

[235] Prasad M, Fine K, Gee A, Nair N, Popp CJ, Cheng B, Manoogian EN, Panda S, Laferrère B (2021). A smartphone intervention to promote time restricted eating reduces body weight and blood pressure in adults with overweight and obesity: a pilot study. Nutrients.

[236] Psihogios AM, King-Dowling S, O'Hagan B, Darabos K, Maurer L, Young J, Fleisher L, Barakat LP, Szalda D, Hill-Kayser CE, Schwartz LA (2021). Contextual predictors of engagement in a tailored mHealth intervention for adolescent and young adult cancer survivors. Ann Behav Med.

[237] Rabbi M, Aung MS, Gay G, Reid MC, Choudhury T (2018). Feasibility and acceptability of mobile phone-based auto-personalized physical activity recommendations for chronic pain self-management: pilot study on adults. J Med Internet Res.

[238] Ramsey SE, Ames EG, Uber J, Habib S, Clark S, Waldrop D (2021). A preliminary test of an mHealth facilitated health coaching intervention to improve medication adherence among persons living with HIV. AIDS Behav.

[239] Reading M, Baik D, Beauchemin M, Hickey K, Merrill J (2018). Factors influencing sustained engagement with ECG self-monitoring: perspectives from patients and health care providers. Appl Clin Inform.

[240] Redfern J, Coorey G, Mulley J, Scaria A, Neubeck L, Hafiz N, Pitt C, Weir K, Forbes J, Parker S, Bampi F, Coenen A, Enright G, Wong A, Nguyen T, Harris M, Zwar N, Chow CK, Rodgers A, Heeley E, Panaretto K, Lau A, Hayman N, Usherwood T, Peiris D (2020). A digital health intervention for cardiovascular disease management in primary care (CONNECT) randomized controlled trial. NPJ Digit Med.

[241] Reyes AT, Bhatta TR, Muthukumar V, Gangozo WJ (2020). Testing the acceptability and initial efficacy of a smartphone-app mindfulness intervention for college student veterans with PTSD. Arch Psychiatr Nurs.

[242] Rico TM, Dos Santos Machado K, Fernandes VP, Madruga SW, Noguez PT, Barcelos CR, Santin MM, Petrarca CR, Dumith SC (2017). Text messaging (SMS) helping cancer care in patients undergoing chemotherapy treatment: a pilot study. J Med Syst.

[243] Rizvi SL, Hughes CD, Thomas MC (2016). The DBT coach mobile application as an adjunct to treatment for suicidal and self-injuring individuals with borderline personality disorder: a preliminary evaluation and challenges to client utilization. Psychol Serv.

[244] Rodriguez Hermosa JL, Fuster Gomila A, Puente Maestu L, Amado Diago CA, Callejas González FJ, Malo De Molina Ruiz R, Fuentes Ferrer ME, Álvarez Sala-Walther JL, Calle Rubio M (2020). Compliance and utility of a smartphone app for the detection of exacerbations in patients with chronic obstructive pulmonary disease: cohort study. JMIR Mhealth Uhealth.

[245] Rosenberger EM, DeVito Dabbs AJ, DiMartini AF, Landsittel DP, Pilewski JM, Dew MA (2017). Long-term follow-up of a randomized controlled trial evaluating a mobile health intervention for self-management in lung transplant recipients. Am J Transplant.

[246] Ross EL, Jamison RN, Nicholls L, Perry BM, Nolen KD (2020). Clinical integration of a smartphone app for patients with chronic pain: retrospective analysis of predictors of benefits and patient engagement between clinic visits. J Med Internet Res.

[247] Rudolf I, Pieper K, Nolte H, Junge S, Dopfer C, Sauer-Heilborn A, Ringshausen FC, Tümmler B, von Jan U, Albrecht U, Fuge J, Hansen G, Dittrich A (2019). Assessment of a mobile app by adolescents and young adults with cystic fibrosis: pilot evaluation. JMIR Mhealth Uhealth.

[248] Rygh P, Asklund I, Samuelsson E (2021). Real-world effectiveness of app-based treatment for urinary incontinence: a cohort study. BMJ Open.

[249] Saberi P, Lisha NE, Erguera XA, Hudes ES, Johnson MO, Ruel T, Neilands TB (2021). A mobile health app (WYZ) for engagement in care and antiretroviral therapy adherence among youth and young adults living with HIV: single-arm pilot intervention study. JMIR Form Res.

[250] Santiago-Torres M, Mull KE, Sullivan BM, Kwon D, Nollen NL, Zvolensky MJ, Bricker JB (2022). Efficacy and utilization of an acceptance and commitment therapy-based smartphone application for smoking cessation among Black adults: secondary analysis of the iCanQuit randomized trial. Addiction.

[251] Santiago-Torres M, Mull KE, Sullivan BM, Kwon DM, Nez Henderson P, Nelson LA, Patten CA, Bricker JB (2022). Efficacy and utilization of smartphone applications for smoking cessation among American Indians and Alaska Natives: results from the iCanQuit trial. Nicotine Tob Res.

[252] Schlosser DA, Campellone TR, Truong B, Anguera JA, Vergani S, Vinogradov S, Arean P (2017). The feasibility, acceptability, and outcomes of PRIME-D: a novel mobile intervention treatment for depression. Depress Anxiety.

[253] Schnall R, Cho H, Mangone A, Pichon A, Jia H (2018). Mobile health technology for improving symptom management in low income persons living with HIV. AIDS Behav.

[254] Schneider T, Baum L, Amy A, Marisa C (2020). I have most of my asthma under control and I know how my asthma acts: users' perceptions of asthma self-management mobile app tailored for adolescents. Health Informatics J.

[255] Scott CK, Dennis ML, Johnson KA, Grella CE (2020). A randomized clinical trial of smartphone self-managed recovery support services. J Subst Abuse Treat.

[256] Selter A, Tsangouri C, Ali SB, Freed D, Vatchinsky A, Kizer J, Sahuguet A, Vojta D, Vad V, Pollak JP, Estrin D (2018). An mHealth app for self-management of chronic lower back pain (Limbr): pilot study. JMIR Mhealth Uhealth.

[257] Seng EK, Prieto P, Boucher G, Vives-Mestres M (2018). Anxiety, incentives, and adherence to self-monitoring on a mobile health platform: a naturalistic longitudinal cohort study in people with headache. Headache.

[258] Serlachius A, Schache K, Kieser A, Arroll B, Petrie K, Dalbeth N (2019). Association between user engagement of a mobile health app for gout and improvements in self-care behaviors: randomized controlled trial. JMIR Mhealth Uhealth.

[259] Serper M, Barankay I, Chadha S, Shults J, Jones LS, Olthoff KM, Reese PP (2020). A randomized, controlled, behavioral intervention to promote walking after abdominal organ transplantation: results from the LIFT study. Transpl Int.

[260] Shaw RJ, Yang Q, Barnes A, Hatch D, Crowley MJ, Vorderstrasse A, Vaughn J, Diane A, Lewinski AA, Jiang M, Stevenson J, Steinberg D (2020). Self-monitoring diabetes with multiple mobile health devices. J Am Med Inform Assoc.

[261] Shebib R, Bailey JF, Smittenaar P, Perez DA, Mecklenburg G, Hunter S (2019). Randomized controlled trial of a 12-week digital care program in improving low back pain. NPJ Digit Med.

[262] Shingleton RM, Pratt EM, Gorman B, Barlow DH, Palfai TP, Thompson-Brenner H (2016). Motivational text message intervention for eating disorders: a single-case alternating treatment design using ecological momentary assessment. Behav Ther.

[263] Siengsukon CF, Silveira Beck Jr E, Drerup M (2021). Feasibility and treatment effect of a web-based cognitive behavioral therapy for insomnia program in individuals with multiple sclerosis: a pilot randomized controlled trial. Int J MS Care.

[264] Signal V, McLeod M, Stanley J, Stairmand J, Sukumaran N, Thompson D, Henderson K, Davies C, Krebs J, Dowell A, Grainger R, Sarfati D (2020). A mobile- and web-based health intervention program for diabetes and prediabetes self-management (BetaMe/Melon): process evaluation following a randomized controlled trial. J Med Internet Res.

[265] Slepian PM, Peng M, Janmohamed T, Kotteeswaran Y, Manoo V, Blades AM, Fiorellino J, Katznelson R, Tamir D, McRae K, Kahn M, Huang A, Kona S, Thaker S, Weinrib A, Katz J, Clarke H (2020). Engagement with manage my pain mobile health application among patients at the transitional pain service. Digit Health.

[266] Spring B, Pellegrini CA, Pfammatter A, Duncan JM, Pictor A, McFadden HG, Siddique J, Hedeker D (2017). Effects of an abbreviated obesity intervention supported by mobile technology: the ENGAGED randomized clinical trial. Obesity (Silver Spring).

[267] St-Jules DE, Woolf K, Goldfarb DS, Pompeii ML, Li H, Wang C, Mattoo A, Marcum ZA, Sevick MA (2021). Feasibility and acceptability of mHealth interventions for managing hyperphosphatemia in patients undergoing hemodialysis. J Ren Nutr.

[268] Steare T, O'Hanlon P, Eskinazi M, Osborn D, Lloyd-Evans B, Jones R, Rostill H, Amani S, Johnson S (2020). Smartphone-delivered self-management for first-episode psychosis: the ARIES feasibility randomised controlled trial. BMJ Open.

[269] Steinert A, Eicher C, Haesner M, Steinhagen-Thiessen E (2020). Effects of a long-term smartphone-based self-monitoring intervention in patients with lipid metabolism disorders. Assist Technol.

[270] Stolz T, Schulz A, Krieger T, Vincent A, Urech A, Moser C, Westermann S, Berger T (2018). A mobile app for social anxiety disorder: a three-arm randomized controlled trial comparing mobile and PC-based guided self-help interventions. J Consult Clin Psychol.

[271] Strauss C, Dunkeld C, Cavanagh K (2021). Is clinician-supported use of a mindfulness smartphone app a feasible treatment for depression? A mixed-methods feasibility study. Internet Interv.

[272] Stubbins R, He T, Yu X, Puppala M, Ezeana CF, Chen S, Valdivia Y Alvarado M, Ensor J, Rodriguez A, Niravath P, Chang J, Wong ST, Patel T (2018). A behavior-modification, clinical-grade mobile application to improve breast cancer survivors' accountability and health outcomes. JCO Clin Cancer Inform.

[273] Su J, Dugas M, Guo X, Gao G (2020). Influence of personality on mHealth use in patients with diabetes: prospective pilot study. JMIR Mhealth Uhealth.

[274] Sundström c, Gajecki M, Johansson M, Blankers M, Sinadinovic K, Stenlund-Gens E, Berman AH (2016). Guided and unguided internet-based treatment for problematic alcohol use - a randomized controlled pilot trial. PLoS One.

[275] Swendeman D, Sumstine S, Aguilar E, Gorbach P, Comulada W, Gelberg L (2021). Feasibility and acceptability of mobile phone self-monitoring and automated feedback to enhance telephone coaching for people with risky substance use: the QUIT-mobile pilot study. J Addict Med.

[276] Talboom-Kamp EP, Verdijk NA, Kasteleyn MJ, Harmans LM, Talboom IJ, Numans ME, Chavannes NH (2017). High level of integration in integrated disease management leads to higher usage in the e-Vita study: self-management of chronic obstructive pulmonary disease with web-based platforms in a parallel cohort design. J Med Internet Res.

[277] Talboom-Kamp EP, Holstege MS, Chavannes NH, Kasteleyn MJ (2019). Effects of use of an eHealth platform e-Vita for COPD patients on disease specific quality of life domains. Respir Res.

[278] Talboom-Kamp EP, Verdijk NA, Kasteleyn MJ, Harmans LM, Talboom IJ, Numans ME, Chavannes NH (2017). Effect of a combined education and eHealth programme on the control of oral anticoagulation patients (PORTALS study): a parallel cohort design in Dutch primary care. BMJ Open.

[279] Tighe J, Shand F, Ridani R, Mackinnon A, De La Mata N, Christensen H (2017). Ibobbly mobile health intervention for suicide prevention in Australian Indigenous youth: a pilot randomised controlled trial. BMJ Open.

[280] Tincopa MA, Lyden A, Wong J, Jackson EA, Richardson C, Lok AS (2022). Impact of a pilot structured mobile technology based lifestyle intervention for patients with nonalcoholic fatty liver disease. Dig Dis Sci.

[281] Tombor I, Beard E, Brown J, Shahab L, Michie S, West R (2019). Randomized factorial experiment of components of the SmokeFree Baby smartphone application to aid smoking cessation in pregnancy. Transl Behav Med.

[282] Tsui JI, Leroux BG, Radick AC, Schramm ZA, Blalock K, Labelle C, Heerema M, Klein JW, Merrill JO, Saxon AJ, Samet JH, Kim TW (2021). Video directly observed therapy for patients receiving office-based buprenorphine - a pilot randomized controlled trial. Drug Alcohol Depend.

[283] Tu YZ, Chang YT, Chiou HY, Lai K (2021). The effects of continuous usage of a diabetes management app on glycemic control in real-world clinical practice: retrospective analysis. J Med Internet Res.

[284] Turner-McGrievy GM, Wilcox S, Boutté A, Hutto BE, Singletary C, Muth ER, Hoover AW (2017). The Dietary Intervention to Enhance Tracking with Mobile Devices (DIET Mobile) study: a 6-month randomized weight loss trial. Obesity (Silver Spring).

[285] Van Blarigan EL, Chan H, Van Loon K, Kenfield SA, Chan JM, Mitchell E, Zhang L, Paciorek A, Joseph G, Laffan A, Atreya CE, Fukuoka Y, Miaskowski C, Meyerhardt JA, Venook AP (2019). Self-monitoring and reminder text messages to increase physical activity in colorectal cancer survivors (Smart Pace): a pilot randomized controlled trial. BMC Cancer.

[286] Van Blarigan EL, Kenfield SA, Chan JM, Van Loon K, Paciorek A, Zhang L, Chan H, Savoie MB, Bocobo AG, Liu VN, Wong LX, Laffan A, Atreya CE, Miaskowski C, Fukuoka Y, Meyerhardt JA, Venook AP (2020). Feasibility and acceptability of a web-based dietary intervention with text messages for colorectal cancer: a randomized pilot trial. Cancer Epidemiol Biomarkers Prev.

[287] Van Tiem J, Moeckli J, Suiter N, Fuhrmeister L, Pham K, Dindo L, Turvey C (2021). "A link to the outside:" patient perspectives on a mobile texting program to improve depression self-management. Patient Educ Couns.

[288] Vorrink S, Huisman C, Kort H, Troosters T, Lammers J (2017). Perceptions of patients with chronic obstructive pulmonary disease and their physiotherapists regarding the use of an eHealth intervention. JMIR Hum Factors.

[289] Wadensten T, Nyström E, Franzén K, Lindam A, Wasteson E, Samuelsson E (2021). A mobile app for self-management of urgency and mixed urinary incontinence in women: randomized controlled trial. J Med Internet Res.

[290] Ware P, Dorai M, Ross HJ, Cafazzo JA, Laporte A, Boodoo C, Seto E (2019). Patient adherence to a mobile phone-based heart failure telemonitoring program: a longitudinal mixed-methods study. JMIR Mhealth Uhealth.

[291] Waselewski ME, Flickinger TE, Canan C, Harrington W, Franklin T, Otero KN, Huynh J, Waldman AL, Hilgart M, Ingersoll K, Ait-Daoud Tiouririne N, Dillingham RA (2021). A mobile health app to support patients receiving medication-assisted treatment for opioid use disorder: development and feasibility study. JMIR Form Res.

[292] Watterson JL, Rodriguez HP, Shortell SM, Aguilera A (2018). Improved diabetes care management through a text-message intervention for low-income patients: mixed-methods pilot study. JMIR Diabetes.

[293] Wei KS, Ibrahim NE, Kumar AA, Jena S, Chew V, Depa M, Mayanil N, Kvedar JC, Gaggin HK (2021). Habits heart app for patient engagement in heart failure management: pilot feasibility randomized trial. JMIR Mhealth Uhealth.

[294] Werner-Seidler A, Wong Q, Johnston L, O'Dea B, Torok M, Christensen H (2019). Pilot evaluation of the Sleep Ninja: a smartphone application for adolescent insomnia symptoms. BMJ Open.

[295] Wright AA, Raman N, Staples P, Schonholz S, Cronin A, Carlson K, Keating NL, Onnela J (2018). The HOPE pilot study: harnessing patient-reported outcomes and biometric data to enhance cancer care. JCO Clin Cancer Inform.

[296] Xu R, Xing M, Javaherian K, Peters R, Ross W, Bernal-Mizrachi C (2020). Improving HbA with glucose self-monitoring in diabetic patients with EpxDiabetes, a phone call and text message-based telemedicine platform: a randomized controlled trial. Telemed J E Health.

[297] Yanez B, Oswald LB, Baik SH, Buitrago D, Iacobelli F, Perez-Tamayo A, Guitelman J, Penedo FJ, Buscemi J (2020). Brief culturally informed smartphone interventions decrease breast cancer symptom burden among Latina breast cancer survivors. Psychooncology.

[298] Yang K, Oh D, Noh JM, Yoon HG, Sun J, Kim HK, Zo JI, Shim YM, Ko H, Lee J, Kim Y (2021). Feasibility of an interactive health coaching mobile app to prevent malnutrition and muscle loss in esophageal cancer patients receiving neoadjuvant concurrent chemoradiotherapy: prospective pilot study. J Med Internet Res.

[299] Yang Q, Hatch D, Crowley MJ, Lewinski AA, Vaughn J, Steinberg D, Vorderstrasse A, Jiang M, Shaw RJ (2020). Digital phenotyping self-monitoring behaviors for individuals with type 2 diabetes mellitus: observational study using latent class growth analysis. JMIR Mhealth Uhealth.

[300] Yang Y, Lee EY, Kim HS, Lee SH, Yoon KH, Cho JH (2020). Effect of a mobile phone-based glucose-monitoring and feedback system for type 2 diabetes management in multiple primary care clinic settings: cluster randomized controlled trial. JMIR Mhealth Uhealth.

[301] Yen S, Ranney ML, Tezanos KM, Chuong A, Kahler CW, Solomon JB, Spirito A (2019). Skills to enhance positivity in suicidal adolescents: results from an open development trial. Behav Modif.

[302] Yingling L, Allen NA, Litchman ML, Colicchio V, Gibson BS (2019). An evaluation of digital health tools for diabetes self-management in Hispanic adults: exploratory study. JMIR Diabetes.

[303] Yoo HJ, Suh EE (2021). Effects of a smartphone-based self-care health diary for heart transplant recipients: a mixed methods study. Appl Nurs Res.

[304] You C, Chen Y, Chen CH, Lee C, Kuo P, Huang M, Chu H (2017). Smartphone-based support system (SoberDiary) coupled with a bluetooth breathalyser for treatment-seeking alcohol-dependent patients. Addict Behav.

[305] Young MD, Morgan PJ (2018). Effect of a gender-tailored eHealth weight loss program on the depressive symptoms of overweight and obese men: pre-post study. JMIR Ment Health.

[306] Zaslavsky O, Thompson HJ, McCurry SM, Landis CA, Kitsiou S, Ward TM, Heitkemper MM, Demiris G (2019). Use of a wearable technology and motivational interviews to improve sleep in older adults with osteoarthritis and sleep disturbance: a pilot study. Res Gerontol Nurs.

[307] Zeng EY, Heffner JL, Copeland WK, Mull KE, Bricker JB (2016). Get with the program: adherence to a smartphone app for smoking cessation. Addict Behav.

[308] Zeng Y, Guo Y, Li L, Hong YA, Li Y, Zhu M, Zeng C, Zhang H, Cai W, Liu C, Wu S, Chi P, Monroe-Wise A, Hao Y, Ho RT (2020). Relationship between patient engagement and depressive symptoms among people living with HIV in a mobile health intervention: secondary analysis of a randomized controlled trial. JMIR Mhealth Uhealth.

[309] Zhang L, He X, Shen Y, Yu H, Pan J, Zhu W, Zhou J, Bao Y (2019). Effectiveness of smartphone app-based interactive management on glycemic control in Chinese patients with poorly controlled diabetes: randomized controlled trial. J Med Internet Res.

[310] Zhang S, Hamburger E, Kahanda S, Lyttle M, Williams R, Jaser SS (2018). Engagement with a text-messaging intervention improves adherence in adolescents with type 1 diabetes: brief report. Diabetes Technol Ther.

[311] Zhang Y, Liu C, Luo S, Huang J, Li X, Zhou Z (2020). Effectiveness of Lilly Connected Care Program (LCCP) app-based diabetes education for patients with type 2 diabetes treated with insulin: retrospective real-world study. JMIR Mhealth Uhealth.

[312] Zhang Y, Liu C, Luo S, Huang J, Yang Y, Ma X, Li X, Zhou Z (2021). Effectiveness of the family portal function on the Lilly Connected Care Program (LCCP) for patients with type 2 diabetes: retrospective cohort study with propensity score matching. JMIR Mhealth Uhealth.

[313] Zheng X, Spatz ES, Bai X, Huo X, Ding Q, Horak P, Wu X, Guan W, Chow CK, Yan X, Sun Y, Wang X, Zhang H, Liu J, Li J, Li X, Spertus JA, Masoudi FA, Krumholz HM (2019). Effect of text messaging on risk factor management in patients with coronary heart disease: the CHAT randomized clinical trial. Circ Cardiovasc Qual Outcomes.

[314] Nguyen E, Bugno L, Kandah C, Plevinsky J, Poulopoulos N, Wojtowicz A, Schneider KL, Greenley RN (2016). Is there a good app for that? Evaluating m-Health apps for strategies that promote pediatric medication adherence. Telemed J E Health.

[315] Carmody JK, Denson LA, Hommel KA (2019). Content and usability evaluation of medication adherence mobile applications for use in pediatrics. J Pediatr Psychol.

[316] Hatem S, Long JC, Best S, Fehlberg Z, Nic Giolla Easpaig B, Braithwaite J (2022). Mobile apps for people with rare diseases: review and quality assessment using mobile app rating scale. J Med Internet Res.

[317] (2003). Adherence to long-term therapies?: evidence for action. World Health Organization.

[318] Röhricht F, Padmanabhan R, Binfield P, Mavji D, Barlow S (2021). Simple mobile technology health management tool for people with severe mental illness: a randomised controlled feasibility trial. BMC Psychiatry.

[319] Gordon K, Dainty KN, Steele Gray C, DeLacy J, Shah A, Resnick M, Seto E (2020). Experiences of complex patients with telemonitoring in a nurse-led model of care: multimethod feasibility study. JMIR Nurs.

[320] Zha P, Qureshi R, Porter S, Chao Y, Pacquiao D, Chase S, O'Brien-Richardson P (2020). Utilizing a mobile health intervention to manage hypertension in an underserved community. West J Nurs Res.

[321] McLeod C, Norman R, Litton E, Saville BR, Webb S, Snelling TL (2019). Choosing primary endpoints for clinical trials of health care interventions. Contemp Clin Trials Commun.

