# Supplementary material for: User Engagement With mHealth Interventions to Promote Treatment Adherence and Self-Management in People With Chronic Health Conditions: Systematic Review
Source: J Med Internet Res. 2024 Sep 24;26:e50508. doi: 10.2196/50508 (PMC11462107; doi:10.2196/50508)
Supplement: Multimedia Appendix 1 [file jmir_v26i1e50508_app1.pdf]

SEARCH STRATEGIES:

Ovid MEDLINE(R) and Epub Ahead of Print, In-Process, In-Data-Review & Other Non-Indexed Citations and Daily <1946 to December 10, 2021>

#	**Query**
1	exp Mobile Applications/ or ((mobile or wearable or smartphone or texting or 'text-messag*' or mhealth or ehealth) adj4 (technology or phone or electronic or platform or app or applicatio* or apps or software or interface or interventio* or coach* or counse* or device or devices or sensor or sensors)).tw.
2	exp "Treatment Adherence and Compliance"/ or Self Care/ or Self-Management/ or (((Drug$ or medication$ or patient$ or client$ or therap* or treatment$) adj4 (complian* or non$complian* or adher* or non$adher* or cooperat* or non$cooperat* or accept* or non$accept* or utilize* or manag*)) or (self adj3 (care or manag*))).tw.
3	Motivation/ or psychology.fx. or (engag* or motiv* or persua* or interac* or experience$ or ((log or logging or logged) adj (in or on))).tw.
4	exp "Evaluation Studies as Topic"/ or (evaluation or pre post tests or pre-post test or pre-post tests or "use effectiveness" or use-effectiveness or (feasibility studies or feasibility study or pilot or program appropriateness or program effectiveness or program sustainability)).tw. or trial.mp. or clinical trial.pt. or random*.mp. or tu.xs.
5	1 and 2 and 3 and 4

Embase

Query('mobile application'/exp OR (((mobile OR wearable OR smartphone OR texting OR 'text-messag*' OR mhealth OR ehealth) NEAR/4 (technology OR phone OR electronic OR platform OR app OR applicatio* OR apps OR software OR interface OR interventio* OR coach* OR counse* OR device OR devices OR sensor OR sensors)):ti,ab,kw)) AND ('patient compliance'/exp OR 'self care'/exp OR ((drug? OR medication? OR patient? OR client? OR therap* OR treatment?) NEAR/4 (complian* OR non?complian* OR adher* OR non?adher* OR cooperat* OR non?cooperat* OR accept* OR non?accept* OR utilize* OR manag*)) OR ((self NEAR/3 (care OR manag*)):ti,ab,kw)) AND ('motivation'/exp OR psychology OR engag*:ti,ab,kw OR motiv*:ti,ab,kw OR persua*:ti,ab,kw OR interac*:ti,ab,kw OR experience?:ti,ab,kw OR (((log OR logging OR logged) NEXT/1 (in OR on)):ti,ab,kw)) AND (evaluation:ti,ab,kw OR 'pre post tests':ti,ab,kw OR 'pre-post test':ti,ab,kw OR 'pre-post tests':ti,ab,kw OR 'use effectiveness':ti,ab,kw OR 'feasibility studies':ti,ab,kw OR 'feasibility study':ti,ab,kw OR pilot:ti,ab,kw OR 'program appropriateness':ti,ab,kw OR 'program effectiveness':ti,ab,kw OR 'program sustainability':ti,ab,kw OR 'evaluation study'/exp OR 'clinical study'/exp)

Web of Science

TS=((mobile OR wearable OR smartphone OR texting OR 'text-messag*' OR mhealth OR ehealth) NEAR/4 (technology OR phone OR electronic OR platform OR app OR applicatio* OR apps OR software OR interface OR interventio* OR coach* OR counse* OR device OR devices OR sensor OR sensors)) AND ((TS=(((Drug? OR medication? OR patient? OR client? OR therap* OR treatment?) NEAR/4 (complian* OR non?complian* OR adher* OR non?adher* OR cooperat* OR non?cooperat* OR accept* OR non?accept* OR utilize* OR manag*)) OR (self NEAR/3 (care OR manag*))))) AND (TS=(Motivation OR psychology) OR (TS=(engag* OR motiv* OR persua* OR interac* OR experience? OR ((log OR logging OR logged) NEAR/0 (in OR on))))) AND (TS=(evaluation OR "pre post tests" OR "pre-post test" OR "pre-post tests" OR "use effectiveness" OR use-effectiveness OR ("feasibility studies" OR "feasibility study" OR pilot OR "program appropriateness" OR "program effectiveness" OR "program sustainability")))

PsycINFO

(DE "Evaluation" OR DE "Needs Assessment" OR DE "Peer Evaluation" OR DE "Personnel Evaluation" OR DE "Program Evaluation" OR ((TI evaluation OR AB evaluation) OR (TI "pre post tests" OR AB "pre post tests") OR (TI "pre-post test" OR AB "pre-post test") OR (TI "pre-post tests" OR AB "pre-post tests") OR (TI "use effectiveness" OR AB "use effectiveness") OR (TI use-effectiveness OR AB use-effectiveness) OR ((TI "feasibility studies" OR AB "feasibility studies") OR (TI "feasibility study" OR AB "feasibility study") OR (TI pilot OR AB pilot) OR (TI "program appropriateness" OR AB "program appropriateness") OR (TI "program effectiveness" OR AB "program effectiveness") OR (TI "program sustainability" OR AB "program sustainability")))) OR TX (trial or random*)

DE "Motivation" OR DE "Achievement Motivation" OR DE "Affiliation Motivation" OR DE "Agency" OR DE "Aspirations" OR DE "Intrinsic Motivation" OR ((TI engag* OR AB engag*) OR (TI motiv* OR AB motiv*) OR (TI persua* OR AB persua*) OR (TI interac* OR AB interac*) OR (TI experience? OR AB experience?) OR (((TI log OR AB log) OR (TI logging OR AB logging) OR (TI logged OR AB logged)) W1 ((TI in OR AB in) OR (TI on OR AB on))))

(DE "Client Attitudes" OR DE "Treatment Compliance") OR (((((TI Drug? OR AB Drug?) OR (TI medication? OR AB medication?) OR (TI patient? OR AB patient?) OR (TI client? OR AB client?) OR (TI therap* OR AB therap*) OR (TI treatment? OR AB treatment?)) N4 ((TI complian* OR AB complian*) OR (TI non?complian* OR AB non?complian*) OR (TI adher* OR AB adher*) OR (TI non?adher* OR AB non?adher*) OR (TI cooperat* OR AB cooperat*) OR (TI non?cooperat* OR AB non?cooperat*) OR (TI accept* OR AB accept*) OR (TI non?accept* OR AB non?accept*) OR (TI utilize* OR AB utilize*) OR (TI manag* OR AB manag*))) OR ((TI self OR AB self) N3 ((TI care OR AB care) OR (TI manag* OR AB manag*)))))

DE "Mobile Applications" OR (((TI mobile OR AB mobile) OR (TI wearable OR AB wearable) OR (TI smartphone OR AB smartphone) OR (TI texting OR AB texting) OR (TI 'text-messag*' OR AB 'text-messag*') OR (TI mhealth OR AB mhealth) OR (TI ehealth OR AB ehealth)) N4 ((TI technology OR AB technology) OR (TI phone OR AB phone) OR (TI electronic OR AB electronic) OR (TI platform OR AB platform) OR (TI app OR AB app) OR (TI applicatio* OR AB applicatio*) OR (TI apps OR AB apps) OR (TI software OR AB software) OR (TI interface OR AB interface) OR (TI interventio* OR AB interventio*) OR (TI coach* OR AB coach*) OR (TI counse* OR AB counse*) OR (TI device OR AB device) OR (TI devices OR AB devices) OR (TI sensor OR AB sensor) OR (TI sensors OR AB sensors)))

CINAHL

(MH "Evaluation Research+") OR (((TI evaluation OR AB evaluation) OR (TI "pre post tests" OR AB "pre post tests") OR (TI "pre-post test" OR AB "pre-post test") OR (TI "pre-post tests" OR AB "pre-post tests") OR (TI "use effectiveness" OR AB "use effectiveness") OR (TI use-effectiveness OR AB use-effectiveness) OR ((TI "feasibility studies" OR AB "feasibility studies") OR (TI "feasibility study" OR AB "feasibility study") OR (TI pilot OR AB pilot) OR (TI "program appropriateness" OR AB "program appropriateness") OR (TI "program effectiveness" OR AB "program effectiveness") OR (TI "program sustainability" OR AB "program sustainability")))) OR TX (trial or random*))

(MH "Motivation+") OR (((TI engag* OR AB engag*) OR (TI motiv* OR AB motiv*) OR (TI persua* OR AB persua*) OR (TI interac* OR AB interac*) OR (TI experience? OR AB experience?) OR (((TI log OR AB log) OR (TI logging OR AB logging) OR (TI logged OR AB logged)) W1 ((TI in OR AB in) OR (TI on OR AB on)))))

((MH "Patient Compliance+") OR (MH "Medication Compliance")) OR (MH "Self Care+") OR ((((((TI Drug? OR AB Drug?) OR (TI medication? OR AB medication?) OR (TI patient? OR AB patient?) OR (TI client? OR AB client?) OR (TI therap* OR AB therap*) OR (TI treatment? OR AB treatment?)) N4 ((TI complian* OR AB complian*) OR (TI non?complian* OR AB non?complian*) OR (TI adher* OR AB adher*) OR (TI non?adher* OR AB non?adher*) OR (TI cooperat* OR AB cooperat*) OR (TI non?cooperat* OR AB non?cooperat*) OR (TI accept* OR AB accept*) OR (TI non?accept* OR AB non?accept*) OR (TI utilize* OR AB utilize*) OR (TI manag* OR AB manag*))) OR ((TI self OR AB self) N3 ((TI care OR AB care) OR (TI manag* OR AB manag*))))))

(MH "Mobile Applications") OR ((((TI mobile OR AB mobile) OR (TI wearable OR AB wearable) OR (TI smartphone OR AB smartphone) OR (TI texting OR AB texting) OR (TI 'text-messag*' OR AB 'text-messag*') OR (TI mhealth OR AB mhealth) OR (TI ehealth OR AB ehealth)) N4 ((TI technology OR AB technology) OR (TI phone OR AB phone) OR (TI electronic OR AB electronic) OR (TI platform OR AB platform) OR (TI app OR AB app) OR (TI applicatio* OR AB applicatio*) OR (TI apps OR AB apps) OR (TI software OR AB software) OR (TI interface OR AB interface) OR (TI interventio* OR AB interventio*) OR (TI coach* OR AB coach*) OR (TI counse* OR AB counse*) OR (TI device OR AB device) OR (TI devices OR AB devices) OR (TI sensor OR AB sensor) OR (TI sensors OR AB sensors))))
