# Supplementary material for: User Engagement With mHealth Interventions to Promote Treatment Adherence and Self-Management in People With Chronic Health Conditions: Systematic Review
Source: J Med Internet Res. 2024 Sep 24;26:e50508. doi: 10.2196/50508 (PMC11462107; doi:10.2196/50508)
Supplement: Multimedia Appendix 3 [file jmir_v26i1e50508_app3.docx]

Supplemental Table 1. mHealth Study Characteristics

| **Characteristic** | **All**  **(n=292)**  **N (%)** | **Adult**  **(n=241)**  **N (%)** | **Pediatric**  **(n=45)**  **N (%)** |
| --- | --- | --- | --- |
| **Health condition** |  |  |  |
| Diabetes | 51 (18) | 40 (17) | 10 (22) |
| Type 2 diabetes | 30 (59) | 30 (75) | 0 |
| Type 1 diabetes | 10 (20) | 0 | 10 (100) |
| Combined diabetes type sample | 11 (21) | 10 (25) | 0 |
| Mental health condition | 35 (12) | 33 (14) | 1 (2) |
| Substance use | 32 (11) | 31 (13) | 1 (2) |
| Cancer | 23 (8) | 21 (9) | 2 (4) |
| Human immunodeficiency virus (HIV) | 18 (6) | 15 (6) | 3 (7) |
| Overweight/obesity | 18 (6) | 15 (6) | 2 (4) |
| Cardiac condition | 16 (6) | 15 (6) | 1 (2) |
| Chronic pain | 14 (5) | 11 (5) | 3 (7) |
| Asthma | 12 (4) | 3 (1) | 8 (18) |
| Chronic obstructive pulmonary disease | 10 (3) | 10 (4) | 0 |
| Hypertension | 9 (3) | 9 (4) | 0 |
| Solid organ transplant | 6 (2) | 4 (2) | 1 (2) |
| Sickle cell disease | 4 (1) | 0 | 4 (9) |
| Chronic kidney disease | 3 (1) | 2 (1) | 1 (2) |
| Urinary incontinence | 3 (1) | 3 (1) | 0 |
| Migraine or headache | 3 (1) | 2 (1) | 1 (2) |
| HIV + substance use | 3 (1) | 3 (1) | 0 |
| Cystic fibrosis | 2 (1) | 0 | 2 (4) |
| Epilepsy | 2 (1) | 0 | 1 (2) |
| Sleep disturbance or insomnia | 2 (1) | 1 (<1) | 1 (2) |
| Parkinson’s disease | 2 (1) | 2 (1) | 0 |
| Other or >1 | 24 (8) | 21 (9) | 3 (7) |
| **Study design** |  |  |  |
| Randomized controlled trial | 135 (46) | 114 (47) | 20 (44) |
| Non-randomized experimental | 116 (40) | 92 (38) | 20 (44) |
| Other | 41 (14) | 35 (15) | 5 (11) |
| **Study location** |  |  |  |
| United States | 165 (57) | 132 (55) | 31 (69) |
| Outside United States | 127 (43) | 109 (45) | 14 (31) |
| **Feasibility study** |  |  |  |
| Yes | 135 (46) | 108 (45) | 24 (53) |
| No | 157 (54) | 133 (55) | 21 (47) |
| **Monetary compensation for participation** |  |  |  |
| Yes | 124 (43) | 95 (39) | 29 (64) |
| No or not reported | 168 (57) | 146 (61) | 16 (36) |

*Note.* There were six studies excluded from the Adult and Pediatric subgroup analysis (five studies included both adult and pediatric participants, one study did not report the sample age). Percentages under “Type 2 diabetes,” “Type 1 diabetes,” and “Combined sample” are calculated as denominator=total Diabetes cases.

Supplemental Table 2. mHealth Intervention Characteristics

| **Characteristic** | **All**  **(N=292)**  **N (%)** | **Adult**  **(n=241)**  **N (%)** | **Pediatric**  **(n=45)**  **N (%)** |
| --- | --- | --- | --- |
| **Intervention adherence and self-management targets** |  |  |  |
| Taking medicine (includes inhalers) | 98 (37) | 70 (29) | 24 (53) |
| Exercise | 93 (32) | 82 (34) | 9 (20) |
| Diet | 73 (25) | 61 (25) | 10 (22) |
| Symptom control | 65 (22) | 51 (21) | 13 (29) |
| Mental health management | 50 (17) | 45 (19) | 4 (9) |
| Drug and/or alcohol use/abuse | 42 (14) | 40 (17) | 2 (4) |
| General self-management and lifestyle modifications | 42 (14) | 31 (13) | 11 (24) |
| Blood glucose monitoring | 38 (13) | 30 (12) | 7 (16) |
| Blood pressure monitoring | 20 (7) | 20 (8) | 0 |
| Weight management outside diet and exercise | 23 (8) | 22 (9) | 0 |
| Sleep | 17 (6) | 11 (5) | 3 (13) |
| Clinic attendance | 11 (4) | 9 (4) | 1 (2) |
| Monitoring other health outcomes | 10 (3) | 8 (3) | 2 (4) |
| Disease knowledge and education | 10 (3) | 9 (4) | 1 (2) |
| Stress management and emotional coping | 7 (2) | 7 (3) | 0 |
| Other | 13 (5) | 12 (5) | 1 (2) |
| **mHealth component** |  |  |  |
| Mobile app (Smartphone or tablet) | 220 (75) | 182 (76) | 33 (73) |
| Text messaging or notification | 74 (25) | 61 (25) | 12 (27) |
| Non-wearable monitoring device (e.g., Bluetooth-enabled pill bottle, blood pressure monitor, glucometer) | 47 (16) | 37 (15) | 8 (18) |
| Website or web portal not within a mobile app | 48 (16) | 44 (18) | 4 (9) |
| Video web-conferencing or telephone | 46 (16) | 42 (17) | 2 (7) |
| Wearable monitoring device (e.g., Fitbit, actigraph) | 39 (13) | 35 (15) | 4 (9) |
| Email | 17 (6) | 15 (6) | 2 (4) |
| Financial incentive tied to use of the technology or meeting mHealth intervention goals | 10 (3) | 6 (3) | 4 (9) |
| Other mHealth component | 9 (3) | 5 (2) | 3 (7) |
| **mHealth user** |  |  |  |
| Patient | 291 (99) | 241 (100) | 45 (100) |
| Caregiver | 20 (7) | 1 (<1) | 17 (38) |
| Healthcare provider | 58 (20) | 48 (20) | 8 (18) |

*Note.* Percentages within headings do not add up to 100% because studies could fall into >1 category. There were six studies excluded from the Adult and Pediatric subgroup analysis (five studies included both adult and pediatric participants, one study did not report the sample age).

Supplemental Table 3. How is user engagement defined?

| **Term** | **All**  **(N=292)**  **N (%)** | **Adult**  **(n=241)**  **N (%)** | **Pediatric**  **(n=45)**  **N (%)** | **Research end point**  **(n=56)**  **N (%)** |
| --- | --- | --- | --- | --- |
| Use (usage, usability, user, utilization) | 102 (35) | 83 (34) | 15 (33) | 16 (29) |
| Engagement (engage, engaged) | 94 (32) | 73 (30) | 19 (42) | 24 (43) |
| Adherence | 59 (20) | 50 (21) | 8 (18) | 16 (29) |
| Feasibility | 43 (15) | 39 (16) | 4 (9) | 8 (14) |
| Acceptability | 26 (9) | 21 (9) | 5 (11) | 6 (11) |
| User experience (experience) | 22 (8) | 21 (9) | 1 (2) | 1 (2) |
| Response(s) | 21 (7) | 14 (6) | 7 (16) | 2 (4) |
| Compliance | 18 (6) | 14 (6) | 2 (4) | 1 (2) |
| Completion | 17 (6) | 17 (7) | 0 | 2 (4) |
| Login (logging, log) | 12 (4) | 9 (4) | 3 (7) | 1 (2) |
| Dose, dosage | 8 (3) | 6 (3) | 2 (4) | 0 |
| Satisfaction | 6 (2) | 6 (3) | 0 | 0 |
| Adoption | 6 (2) | 4 (2) | 2 (4) | 1 (2) |
| Perception(s) | 6 (2) | 5 (2) | 1 (2) | 0 |
| Access | 5 (2) | 3 (1) | 2 (4) | 0 |
| Data | 5 (2) | 5 (2) | 0 | 2 (4) |
| Fidelity | 5 (2) | 3 (1) | 2 (4) | 0 |
| Interact (interactive) | 5 (2) | 4 (2) | 0 | 0 |
| Participation | 4 (1) | 4 (2) | 0 | 1 (2) |
| Retention | 4 (1) | 4 (2) | 0 | 2 (4) |
| Self-monitor (monitoring) | 4 (1) | 3 (1) | 1 (2) | 1 (2) |
| Implementation | 3 (1) | 1 (<1) | 2 (4) | 0 |
| Uptake | 2 (<1) | 1 (<1) | 1 (2) | 0 |
| Registration | 2 (<1) | 1 (<1) | 1 (2) | 0 |
| Track (tracked) | 2 (<1) | 1 (<1) | 0 | 0 |
| Maintenance | 2 (<1) | 0 | 2 (4) | 0 |
| Tolerability | 2 (<1) | 2 (<1) | 0 | 1 (2) |
| Enter (entries) | 2 (<1) | 2 (<1) | 0 | 0 |
| Followed | 2 (<1) | 2 (<1) | 0 | 0 |
| Reading | 2 (<1) | 1 (<1) | 1 (2) | 1 (2) |
| Sync (synchronization) | 2 (<1) | 1 (<1) | 1 (2) | 1 (2) |
| Install, download | 2 (<1) | 2 (<1) | 0 | 0 |
| Wear (wore) | 2 (<1) | 2 (<1) | 0 | 1 (2) |
| Other terms | 30 (11) | 25 (10) | 4 (9) | 5 (9) |

*Note. “*Other terms” was assigned to terminology appearing in 1 study (<1%) in the overall sample. Percentages within headings do not add up to 100% because studies could fall into >1 category. There were six studies excluded from the Adult and Pediatric subgroup analysis (five studies included both adult and pediatric participants, one study did not report the sample age).

Supplemental Table 4. User engagement evaluation methods in the overall study sample and adult and pediatric only studies

| **User Engagement Evaluation Methods** | **All**  **(n=292)**  **N (%)** | **Adult**  **(n=241)**  **N (%)** | **Pediatric (n=45)**  **N (%)** |
| --- | --- | --- | --- |
| **Objective measures** |  |  |  |
| User login data retrieved from app or website | 160 (55) | 131 (54) | 25 (56) |
| Manual user data entry in app/website-based self-monitoring diaries | 77 (26) | 66 (27) | 9 (20) |
| Response to text messages or push notifications | 49 (17) | 41 (17) | 7 (16) |
| Number or proportion of intervention program modules completed within app/website | 48 (16) | 40 (17) | 6 (13) |
| Interacting via chats, phone calls, or social media posts | 33 (11) | 32 (13) | 1 (2) |
| Wearing an electronic monitoring device | 26 (9) | 24 (10) | 2 (4) |
| Using a non-wearable electronic monitoring device | 26 (9) | 20 (8) | 5 (11) |
| Submitting videos via app | 5 (2) | 4 (2) | 1 (2) |
| Other objective measure | 13 (5) | 10 (4) | 2 (4) |
| **Subjective measures** |  |  |  |
| Qualitative interview | 54 (19) | 45 (19) | 9 (20) |
| Participant-reported survey | 29 (10) | 22 (9) | 7 (16) |

*Note.* Percentages within headings do not add up to 100% because studies could fall into >1 category. There were six studies excluded from the Adult and Pediatric subgroup analysis (five studies included both adult and pediatric participants, one study did not report the sample age). Glucometer was sub-coded within Non-wearable electronic monitoring device.

Supplemental Table 5. Measurement approaches used in interventions containing specific mHealth components

|  | **Mobile app**  **n=220** | **Text messaging**  **n=74** | **Non-wearable device**  **n=47** | **Wearable device**  **n=39** | **Video web conferencing**  **n=46** | **Website**  **n=48** | **Email**  **n=17** | **Financial incentive**  **n=5** | **Other mHealth component**  **n=9** |
| --- | --- | --- | --- | --- | --- | --- | --- | --- | --- |
|  | **n (%)** | **n (%)** | **n (%)** | **n (%)** | **n (%)** | **n (%)** | **n (%)** | **n (%)** | **n (%)** |
| User login data | 138 (63) | 21 (28) | 26 (55) | 17 (44) | 22 (48) | 30 (63) | 7 (41) | 5 (100) | 4 (44) |
| Manual user data entry | 68 (31) | 12 (16) | 15 (32) | 10 (26) | 10 (22) | 10 (21) | 3 (18) | 2 (20) | 1 (11) |
| Response to text messages or push notifications | 16 (7) | 38 (51) | 5 (11) | 5 (13) | 10 (22) | 8 (17) | 0 | 3 (30) | 3 (33) |
| Module completion | 36 (16) | 5 (7) | 4 (9) | 2 (5) | 7 (15) | 17 (35) | 5 (29) | 0 | 1 (11) |
| Interacting via chats, phone calls, or social media posts | 27 (12) | 10 (14) | 5 (11) | 5 (13) | 12 (26) | 6 (13) | 4 (24) | 0 | 0 |
| Wearing an electronic monitoring device | 18 (8) | 10 (14) | 6 (13) | 26 (67) | 10 (22) | 4 (8) | 2 (12) | 2 (20) | 1 (11) |
| Using a non-wearable electronic monitoring device | 24 (11) | 4 (5) | 25 (53) | 6 (15) | 4 (9) | 2 (4) | 0 | 3 (30) | 0 |
| Submitting videos via app | 4 (2) | 2 (3) | 1 (2) | 0 | 0 | 0 | 1 (6) | 3 (30) | 1 (11) |
| Qualitative interview | 36 (16) | 14 (19) | 10 (21) | 5 (13) | 9 (20) | 10 (21) | 2 (12) | 2 (20) | 1 (11) |
| Survey | 25 (11) | 6 (8) | 3 (6) | 2 (5) | 2 (4) | 2 (4) | 1 (6) | 0 | 2 (22) |
| Other | 7 (3) | 6 (8) | 0 | 2 (5) | 1 (2) | 1 (2) | 1 (6) | 0 | 3 (33) |

Supplemental Table 6. User engagement evaluation methods in studies designating user engagement as a research end point

| **User Engagement Evaluation Method** | **Research End Point**  **(n=56)** |
| --- | --- |
| User login data retrieved from app or website | 31 (55) |
| Manual user data entry in app/website-based self-monitoring diaries | 22 (39) |
| Response to text messages or push notifications | 7 (13) |
| Number or proportion of intervention program modules completed within app/website | 5 (9) |
| Interacting via chats, phone calls, or social media posts | 5 (9) |
| Wearing an electronic monitoring device | 8 (14) |
| Using a non-wearable electronic monitoring device | 7 (13) |
| Submitting videos via app | 2 (4) |
| Other objective measures | 2 (4) |
| Qualitative interview | 10 (18) |
| Participant-reported survey | 5 (9) |

*Note*. Percentages do not add up to 100% because studies could fall into >1 category.

Supplemental Table 7. User engagement evaluation methods in studies with and without monetary compensation

|  | **Yes, monetary compensation**  **(n=124)** | **No monetary compensation or not reported**  **(n=168)** |
| --- | --- | --- |
| **User Engagement Evaluation Method** |  |  |
| User login data retrieved from app or website | 70 (56) | 90 (54) |
| Manual user data entry in app/website-based self-monitoring diaries | 28 (23) | 49 (29) |
| Response to text messages or push notifications | 24 (19) | 25 (15) |
| Number or proportion of intervention program modules completed within app/website | 16 (13) | 32 (19) |
| Interacting via chats, phone calls, or social media posts | 14 (11) | 19 (11) |
| Wearing an electronic monitoring device | 12 (10) | 14 (8) |
| Using a non-wearable electronic monitoring device | 9 (7) | 17 (10) |
| Submitting videos via app | 5 (4) | 0 |
| Other objective measures | 6 (5) | 7 (4) |
| Qualitative interview | 27 (22) | 27 (16) |
| Participant-reported survey | 15 (12) | 14 (8) |
| **Level of Engagement** |  |  |
| Low | 14 (11) | 13 (7) |
| Medium | 4 (3) | 2 (1) |
| High | 30 (24) | 33 (20) |
| >1 | 39 (31) | 60 (36) |
| Not characterized | 37 (30) | 60 (36) |

*Note*. For User Engagement Evaluation Method, percentages do not add up to 100% because studies could fall into >1 category.
